# Calcium Dynamics of *Ex Vivo* Long-Term Cultured CD8^+^ T Cells Are Regulated by Changes in Redox Metabolism

**DOI:** 10.1371/journal.pone.0159248

**Published:** 2016-08-15

**Authors:** Catherine A. Rivet, Ariel S. Kniss-James, Margaret A. Gran, Anish Potnis, Abby Hill, Hang Lu, Melissa L. Kemp

**Affiliations:** 1 School of Electrical and Computer Engineering, Georgia Institute of Technology, Atlanta, Georgia, United States of America; 2 The Wallace H. Coulter Department of Biomedical Engineering, Georgia Institute of Technology and Emory University, Atlanta, Georgia, United States of America; 3 School of Chemical and Biomolecular Engineering, Georgia Institute of Technology, Atlanta, Georgia, United States of America; 4 The Parker H. Petit Institute for Bioengineering and Bioscience, Atlanta, Georgia, United States of America; Georgia Regents University Cancer Center, UNITED STATES

## Abstract

T cells reach a state of replicative senescence characterized by a decreased ability to proliferate and respond to foreign antigens. Calcium release associated with TCR engagement is widely used as a surrogate measure of T cell response. Using an ex vivo culture model that partially replicates features of organismal aging, we observe that while the amplitude of Ca^2+^ signaling does not change with time in culture, older T cells exhibit faster Ca^2+^ rise and a faster decay. Gene expression analysis of Ca^2+^ channels and pumps expressed in T cells by RT-qPCR identified overexpression of the plasma membrane CRAC channel subunit ORAI1 and PMCA in older T cells. To test whether overexpression of the plasma membrane Ca^2+^ channel is sufficient to explain the kinetic information, we adapted a previously published computational model by Maurya and Subramaniam to include additional details on the store-operated calcium entry (SOCE) process to recapitulate Ca^2+^ dynamics after T cell receptor stimulation. Simulations demonstrated that upregulation of ORAI1 and PMCA channels is not sufficient to explain the observed alterations in Ca^2+^ signaling. Instead, modeling analysis identified kinetic parameters associated with the IP_3_R and STIM1 channels as potential causes for alterations in Ca^2+^ dynamics associated with the long term ex vivo culturing protocol. Due to these proteins having known cysteine residues susceptible to oxidation, we subsequently investigated and observed transcriptional remodeling of metabolic enzymes, a shift to more oxidized redox couples, and post-translational thiol oxidation of STIM1. The model-directed findings from this study highlight changes in the cellular redox environment that may ultimately lead to altered T cell calcium dynamics during immunosenescence or organismal aging.

## Introduction

Calcium release is an essential step in T cell activation and regulates diverse cellular functions, such as proliferation, apoptosis, differentiation, effector function and gene transcription [1]. After T cell receptor ligation, phosphorylation of phospholipase C-γ (PLCγ) leads to IP_3_ formation and rapid Ca^2+^ release from the ER stores through the IP_3_ receptor channels. T cells sustain elevated cytoplasmic Ca^2+^ levels for gene transcription, by balancing store-operated Ca^2+^ entry (SOCE) through the plasma membrane and Ca^2+^ buffering by the mitochondria. Calcium dynamics encode information from the antigenic peptide:TCR interaction for instructing T cells to activate cytokine production, such as IFN-γ [2].

T cell responses from aged donors are typically slower and of lower amplitude than those from younger individuals, whether the response is measured in terms of cytokine production [3], gene activation for cell cycle entry and transcription [4,5] or activation of protein kinase pathways [6]. We have shown, along with other research groups, that the kinase activation upstream of Ca^2+^ release from the ER stores are downregulated with time in culture [6,7] which would suggest reduced Ca^2+^ signaling; however the literature is conflicted regarding the consequences of age on calcium mobilization. Although Ca^2+^ mobilization has been shown to be impaired in old mice for both CD4^+^ and CD8^+^ T cell subsets [8–10], in humans, CD8^+^ T cells from elderly donors had a slightly greater Ca^2+^ response to stimulation than CD4^+^ cells but a larger reduction in their proliferative potential [11]. Similarly, reports of baseline Ca^2+^ levels in healthy elderly subjects have been controversial, with reports of unchanged [12] or reduced [13] basal Ca^2+^ levels. Induction of a sustained Ca^2+^ signal is critical for CD8^+^ T cell effector function [14,15] and downstream gene regulation through the NFAT pathway; therefore a strong Ca^2+^signal is required for an efficient tumor-specific immune response in the context of adoptive T cell transfer. The differences between murine models and human aging suggest that the effects of *ex vivo* aging on Ca^2+^ signaling, and in particular culture conditions consistent with adoptive cell therapy, may not be intuitive.

Comprehensive microarray studies have been conducted to compare gene expression profiles in T cells between young and old human subjects [4,16]. These studies report the differential expression of several key redox regulatory genes associated with oxidative stress. Age-dependent increases in the levels of lipid peroxidation and protein oxidation, and declines in glutathione levels and activities of antioxidant enzymes in mixed human T cell populations have also been reported[17]. Reactive oxygen species (ROS) are generated by the mitochondria due to metabolism and NADPH oxidases during signaling, but can be effectively eliminated by cellular antioxidant defense mechanisms. Although T cells modulate their redox status for signaling purposes[18], excessive production of ROS can overwhelm the antioxidant defense system, leading to oxidative stress, improper signaling and tissue and DNA damage. These studies suggest an oxidative shift in redox potential *in vivo* as a function of organism age alters T cell signaling.

A wealth of biochemical studies point to sensitivity to oxidation among proteins responsible for intracellular calcium levels including ER receptors and membrane channels, albeit to different degrees and with different functional consequences[19]. For example, OraI1 has been reported to be inhibited in the presence of H_2_O_2_ while Orai3 is oxidation-independent [20]. A redox sensitive cysteine thiol on STIM1 has been identified which lowers the affinity for Ca^2+^ [21]. In addition, the proximity of ER oxidase Ero1α to IP3R receptors, for example, is likely to serve as a local source of catalytic redox regulation under conditions of cellular stress [19]. Despite an indisputable link between cellular redox state and Ca^2+^, many in vitro findings are often performed at levels unattainable in vivo. For example, the induction of store-operated calcium entry in Jurkat cells by exogenous H_2_O_2_ identified IP3R as susceptible to oxidation and TPRM2 as insensitive to oxidation[22], but these conditions may not be fully applicable to the subtleties of aging-related oxidation within intact cells. The physiological oxidative burst in response to TCR engagement may differ in concentration, localization, and timing, when compared to treatment by bolus H_2_O_2_.

Using *ex vivo* replicative senescence as a model for *in vivo* aging and immunosenescence, we sought to determine what systemic effects the long term culture of cytotoxic CD8^+^ T cells have on Ca^2+^ dynamics and used a computational model to inform biochemical experiments to isolate mechanisms responsible for the observed differences. We report a pro-oxidative shift in redox metabolism and associated post-translational changes to thiols of STIM1 that may contribute to observed kinetic changes in calcium handling of CD8^+^ T cells with ex vivo expansion.

## Methods

### Primary CD8^+^ T cell isolation

The Georgia Institute of Technology IRB specifically approved this study under protocol H10253 with written consent of the donors for collection of blood. CD8^+^ T cells were obtained from healthy blood donors (21–35 years old) using standard isolation procedures. Briefly, 40 mL of fresh blood was collected in EDTA coated tubes. Peripheral blood mononuclear cells were isolated by density centrifugation using Lymphoprep (VWR), and CD8^+^ T cells further purified using the Dynabeads® Untouched™ Human CD8 T Cells isolation kit (Invitrogen) (>95% purity as checked by flow cytometry).

### Cell culture and expansion

Jurkat cells (ATCC) were cultured under standard cultured conditions, in RPMI 1640 medium with L-glutamine (Sigma-Aldrich) with 10 mM HEPES, 1 mM sodium pyruvate, and 1X MEM nonessential amino acids, and 50 U/mL penicillin/streptomycin (Cellgro) and 10% certified heat-inactivated fetal bovine serum (Sigma-Aldrich). For primary CD8^+^ T cells, the culture medium was supplemented with 50 U/mL of recombinant IL-2 (Sigma-Aldrich) and Dynabeads® Human T-Activator CD3/CD28 (Invitrogen) at 1:1 bead to cell ratio (kept constant for the entire culture period) for rapid cell expansion [23,24]. Cell cultures were checked daily and resuspended in fresh medium when needed. Expansion beads were removed between 10–12 hours before the beginning of an experiment.

### Dynamic measurements of TCR-induced cytosolic Ca^2+^

Jurkat cells were incubated in phenol red free RPMI 1640 medium with 5 μM Fura Red, 3μM Fluo-3 AM (Molecular Probes) and 0.05% Pluronic F127 for 40 minutes at 37°C, washed three times with cold PBS and resuspended in warm phenol red free medium in the presence or absence of specific chemical inhibitors (30 min pretreatment at the appropriate concentration). Cell fluorescence was read on a BD LSR II flow cytometer using 488 nm laser excitation with FITC and PE filters for Fluo3 and Fura Red fluorescence detection. First, 3 minutes of baseline was obtained. To activate the calcium signaling pathway, 2μg/mL anti-CD3 (clone OKT3) and 2μg/mL anti-CD28 antibodies were added to the cells, then fluorescence was read for 30 additional minutes. Ionomycin was used as positive control and EGTA as a negative control in independent samples to establish the maximum and minimum calcium mobilization signal associated with the cell dye loading protocol.

Primary CD8^+^ T cells were preincubated at 37°C with 3μM Fluo3 AM, 5 μM Fura Red and 0.05% Pluronic F127 for 20 minutes followed by a wash step with cold PBS. Cell were resuspended in cold PBS with 2μg/mL anti-CD3 (clone OKT3) and 2μg/mL anti-CD28 for 30 minutes at 4°C. Fluorescence on the flow cytometer was read for 3 minutes to acquire the Ca^2+^ baseline. To activate the TCR pathway, cells were diluted 10 times in a 37°C phenol-free media containing 20 μg/mL anti-Mouse IgG for rapid crosslinking. Cell fluorescence was recorded for an additional 30 minutes. The median ratio of Fluo3/Fura Red fluorescence (FITC/PE filters) was used to generate relative [Ca^2+^]_i_ traces using the kinetic module of FlowJo (Ashland, OR). Consistent instrument voltage settings were used throughout data collection. All Ca^2+^ traces were first registered to ensure stimulation occurred at the same time. Time courses were smoothed using Savitzky-Golay filtering and normalized to the baseline (0–180 seconds) fluorescence. Peak time, peak amplitude and integral under the curve were calculated using custom Matlab scripts (R2014b (Mathworks, Natick, MA)). Decay parameters were obtained by fitting the decay portion of the dynamics to a sum of exponentials:

$$Decay=A_{1}e^{\frac{t}{\tau_{1}}}+A_{2}e^{\frac{t}{\tau_{2}}}$$ (eqn 1)

### Real-time quantitative reverse transcriptase PCR

Total RNA from CD8^+^ T cells was extracted using the RNeasy Mini isolation kit (SABiosciences, Frederick, MD) with RNase-free DNase set (Qiagen, Valencia, CA) according to the manufacturer's protocol. The integrity and concentration of intact total RNA was verified with a NanoDrop 1000 Spectrophotometer (Thermo Scientific). Real-time PCR was performed with a StepOnePlus RT-qPCR system instrument (Applied Biosystems, Carlsbad, CA) using predesigned gene-specific primer and probe sets (SA Biosciences) for Actin, ORAI1, SERCA2b, SERCA3, PMCA, IP3R2 and IP3R3 or the Human Oxidative Stress and Antioxidant Defense PCR Array containing 84 predesigned gene-specific primer and probe sets following the manufacturer’s protocols. Briefly, 1 μg of total RNA was reverse transcribed and amplified using the RT-PCR kit (Qiagen) following the manufacturer’s instructions. Each 20 μL reaction mixture aliquot contained 1 μL of primer mixture (SA Biosciences), 2 μL of universal PCR Master Mix (Qiagen) and 1–4 μL of cDNA or water as a negative control. Initial denaturation of DNA was carried out at 95°C for 10 min. Forty amplification cycles were performed, each cycle consisting of denaturation (95°C, 30 s) and annealing and extension (65°C, 1 min). Relative expression levels were calculated using the ΔCT method (2^-ΔCT^). For individual calcium gene probe sets, each sample was amplified in triplicate and results were normalized using the housekeeping gene actin [25]. For the oxidative stress arrays, two individual arrays were performed for each donor, one for young cells and one for older cells and normalized to the geometric mean of housekeeping genes GAPDH and HPRT1. Paired t-tests were performed for each normalized target for each donor to determine significant changes in expression between young and aged cells.

### Redox western blotting

For western blotting, 8 × 10^6^ cells were lysed in 100 μl 2% NP-40-based lysis buffer for standard SDS-PAGE analysis or were lysed in a guanidine HCl lysis buffer with 9.3 mg/mL iodoacteic acid (IAA) as previously described for redox western blotting [26]. Bands were normalized to β-actin levels (Sigma Aldrich) as a loading control. Primary antibody for Duox1 was purchased from Novus Biologicals and for Trx1 from Sekisui Diagnostics.

### Measurement of intracellular GSH and GSSG

Glutathione (GSH) and glutathione disulfide (GSSG) were measured by HPLC as S-carboxmethyl N-dansyl derivatives using γ-glutamylglutamate as an internal standard [27].

### Measurement of cellular redox potential

Cellular redox potential with respect to glutathione and thioredoxin was calculated using the Nernst equation: $E_{GSH}=E_{GSH}^{0}-(\frac{RT}{zF})\log\frac{{\left[GSH\right]}^{2}}{\left[GSSG\right]}$ for glutathione and $E_{TRX}=E_{TRX}^{0}-(\frac{RT}{zF})\log\frac{\left[{TRX}_{red}\right]}{\left[{TRX}_{ox}\right]}$ for thioredoxin, where R is the gas constant (9.315 J K^-1^ mol^-1^), T = 298.15 K, z the number of transferred electrons (2), and F is the Faraday constant (96.485 C mol^-1^). The standard redox potentials of GSH and Trx at pH 7 used for the calculations were $E_{GSH}^{0}=-264\;mV$ and $E_{TRX}^{0}=-254\;mV$.

### Measurement of reversible oxidation of STIM1

Primary human CD8^+^ T cells were isolated from whole blood and cultured as described. After 5 and 21 days in culture, primary CD8^+^ T cells were lysed at room temperature in degassed RIPA lysis buffer containing iodoacetamide (100 mM iodoacetamide, 25 mM Tris-HCl, 150 mM NaCl, 10% glycerol, 1% Igepal, 1% sodium deoxycholate, 10 μg/mL aprotinin, 10 μg/mL leupeptin, 1 μg/mL pepstatin, 1 μg/mL microcystin, 200 μM benzamidine). After lysing for 20 min in the dark, the lysates were sonicated on ice for 10 min. Cellular debris was removed by centrifugation at 10000 x g for 20 min at 4°C. The lysates were passed through a Zeba spin desalting column (Pierce) to remove excess iodoacetamide. Samples were then frozen so that all further assay steps could be performed on young and old cells simultaneously. The lysates were pre-cleared with PureProteome Protein A magnetic beads (Millipore) for 30 min at room temperature. STIM1 was immunoprecipitated overnight at 4°C using 1:40 dilution of anti-STIM1 (#4916, Cell Signaling) and subsequently pulled down for 30 min at room temperatures. Beads were washed three times with lysis buffer without iodoacetamide. To reduce oxidized thiols and elute STIM1, lysis buffer containing 1 mM DTT and 2% SDS were added to the IP beads. The samples were heated for 15 min at 37°C and then for 10 min at 90°C. The supernatant was separated from the beads and nascent thiols were biotinylated using 3 mM of PEO-biotin-iodoacetamide (BIAM) (Cyanogen) in the dark at room temperature for 1 h. The reaction was quenched with the addition of 4X reducing Laemmlis sample buffer. For assay validation and controls, Jurkat cells were used instead of primary cells. Oxidized and reduced assay controls were prepared by either lysing in buffer without iodoacetamide or omitting DTT from the elution buffer, respectively. STIM1 oxidation was analyzed by standard Western blotting techniques. STIM1 was detected with (1:1000 anti-STIM1, #4916 Cell Signaling). To detect oxidized cysteines, blots were stripped, blocked overnight at 4°C with Odyssey blocking buffer, and probed with 1:10000 IRDye® 800CW streptavidin (LI-COR Biotechnology) in Rockland blocking buffer for 1 h at room temperature. Blots were imaged with an Odyssey Scanner (LI-COR Biotechnology) and were quanitified in ImageStudio with the background calculated on a lane-by-lane basis according to standard procedures.

### Computational model of calcium dynamics in T lymphocytes

#### Model description

The binding of a peptide/MHC complex to the TCR triggers the recruitment of tyrosine kinases Lck, ITK and Zap70 to the TCR/CD3 complex, ultimately resulting in the phosphorylation and activation of PLC-γ The model represents these receptor-initiated events as a one-step input to phospho-PLC-γ levels. Activated PLC-γ cleaves PIP_2_ in the plasma membrane to generate diacylglycerol (DAG) and 1, 4, 5-inositol triphosphate (IP_3_). Binding of IP_3_ to the IP_3_ receptor (IP_3_R) triggers the release of Ca^2+^ stored in the ER (J_IP3_). The resulting drop in ER Ca^2+^ levels activates the ER Ca^2+^ sensor STIM1, which translocates to the ER-PM (plasma membrane) junctions to activate a more sustained influx in the cytosol through the calcium release activated Ca^2+^ channels (CRAC) on the PM (J_crac_) [28,29]. The PM Ca^2+^ ATP-ase (PMCA) pumps Ca^2+^ out of the cytosol and maintains a steep gradient of Ca^2+^ concentration from 50 nM inside the cell to 1.5 mM in the extracellular space (J_pmca_). Because of this steep gradient, we assume there is a very small Ca^2+^ leak into the cytosol from the extracellular space (J_PMleak_). The Sarco/ER Ca^2+^ ATP-ase (SERCA) pumps cytosolic Ca^2+^ back in the ER stores to maintain an ER luminal concentration of 350 μM (J_serca_). Similarly, we consider a small leak of Ca^2+^ ions from the ER to the cytosol (J_ERleak_). Mitochondria are essential for the activation and maintenance of the store-operated calcium entry (SOCE) by buffering Ca^2+^ ions and preventing the negative feedback of Ca^2+^ on the CRAC channels [30]. Uptake of Ca^2+^ ions in the mitochondria is mediated through the Ca^2+^ uniporter (J_mitin_) and extrusion through the Na^+^/Ca^2+^ exchanger (J_mitout_). The fundamental equations of Ca^2+^ kinetics in the various cellular compartments are described as follows:

$$\frac{d{Ca}_{cyt}}{dt}=\beta_{i}\;((J_{IP3}-J_{serca}+J_{ERleak})+({-J}_{mitin}+J_{mitout})+{\;(J}_{crac}-J_{pmca}+J_{PMleak})$$ (eqn 2)

$$\frac{d{Ca}_{ER}}{dt}=\frac{\beta_{er}}{\rho_{er}}(-J_{IP3}+J_{serca}-J_{ERleak})$$ (eqn 3)

$$\frac{d{Ca}_{mit}}{dt}=\frac{\beta_{mit}}{\rho_{mit}}\;(J_{mitin}-J_{mitout})$$ (eqn 4)

Ca_cyt_, Ca_mit_ and Ca_ER_ denote the concentration of free Ca^2+^ in the cytosol, mitochondria and ER respectively. *β*_*i*_, *β*_*er*_, *β*_*mit*_ are the ratio of free to total Ca^2+^, assuming fast buffering with calcium-binding proteins in the cytosol, ER and mitochondria respectively [31,32]. In this model, we assume that the ratio of free to total Ca^2+^ is constant in the three cellular compartments and do not model explicitly the dynamics of free calcium-binding proteins. *ρ*_*er*_, *ρ*_*mit*_ are the ratios of the ER and mitochondria volume to that of the cytosol.

#### IP_3_ production

Initiation of Ca^2+^ signaling after TCR binding requires formation of IP_3_ through PLC-γ phosphorylation. We modeled PLC-γ activation as a simplified one step mass action kinetics (Eq 6) following ligand (*R*) unbinding from the TCR (Eq 5):

$$\frac{dR}{dt}=-k_{PLCact}∙R$$ (eqn 5)

$$\frac{dpPLC\gamma }{dt}=k_{PLCact}∙R-k_{PLCdeact}∙pPLC\gamma$$ (eqn 6)

where *k*_*PLCact*_ is the rate constant for PLC-γ phosphorylation and *k*_*PLCdeact*_ the rate constant for PLC-γ dephosphorylation.

The production of IP_3_ depends on the levels of phosphorylated PLC-γ and cytoplasmic Ca^2+^ levels, creating a positive feedback enhancing IP_3_ formation:

$$\frac{dIP3}{dt}=k_{IP3prod}∙pPLC\gamma ∙{Ca}_{cyt}-k_{IP3deg}∙IP3$$ (eqn 7)

where *k*_*IP*3*prod*_ is the rate constant for IP_3_ production and *k*_*IP*3*deg*_ the rate constant for IP_3_ degradation.

#### Ca^2+^ flux through the IP3R

IP_3_R is a tetramer of four identical subunits. Each unit has one IP_3_ binding site and two Ca^2+^ binding sites, one for activation and one for inhibition. The channel activity is cooperatively regulated by binding/unbinding of IP_3_ and Ca^2+^ at these binding sites. A number of mathematical models of IP_3_R activation have been constructed, including Bezprozvanny *et al*. [33], De Young and Keizer [34], Atri *et al*. [35], Li and Rinzel [36], Sneyd *et al*. [37]. In these models, the IP_3_R is assumed to be modulated by cytosolic Ca^2+^ in a biphasic manner with Ca^2+^ release inhibited at low and high cytosolic Ca^2+^ levels, and facilitated by intermediate levels. We used the Li-Rinzel description of the IP_3_R [36]. The flux of Ca^2+^ through the IP_3_R is given by:

$$J_{IP3}=V_{IP3}∙P_{IP3}∙{Ca}_{ER}$$ (eqn 8)

where *V*_*IP3*_ is the maximum flowrate, and P_IP3_ is the IP_3_R open probability. P_IP3_ is assumed to be an instantaneous function of Ca^2+^, IP_3_ concentration and the fraction of IP_3_R not inactivated by Ca^2+^ bound to the inhibitory site, *h*. P_IP3_ is described as:

$$P_{IP3}={\left((\frac{IP3}{IP3+K_{IP3}})(\frac{{Ca}_{cyt}}{{Ca}_{cyt}+K_{act}})h\right)}^{3}$$ (eqn 9)

where *K*_*IP*3_ is the concentration of IP_3_ at which the half maximal observed reaction rate is achieved and *K*_*act*_ is midpoint of calcium-dependent channel activation.

The fraction of inactivated IP_3_R (1-*h*), is a function of cytoplasmic Ca^2+^ and *Q*, the effective affinity of Ca^2+^ to the inhibitory site.

$$\frac{dh}{dt}=A((1-hAQ+{Ca}_{cyt})-{Ca}_{cyt})$$ (eqn 10)

$$Q=K_{inh}\left(\frac{IP3+K_{IP3}}{IP3+K_{IP3inh}}\right)$$ (eqn 11)

where *A* is a variable controlling the relative time scales between the differential equations, *K*_*inh*_, the Ca^2+^ affinity to the Ca^2+^ inhibitory site and *K*_*IP*3*inh*_ the affinity of IP_3_ to the IP_3_ binding site when the Ca^2+^ inhibitory site is occupied.

#### Ca^2+^ leak from the ER

Because of the gradient of concentration between the ER and the cytosol, there is a constant leakage of Ca^2+^ ions from the ER to the cytoplasm. *J*_*ERleak*_ can be described as:

$$J_{ERleak}=K_{ERleak}∙Ca_{ER}$$ (eqn 12)

### Ca^2+^ flux through the SERCA pumps

$$J_{serca}=V_{serca}∙\frac{{Ca_{cyt}}^{2}}{{Ca_{cyt}}^{2}+{K_{serca}}^{2}}$$ (eqn 13)

where *V*_*serca*_ is the maximum flux across the SERCA pump and *K*_*serca*_ is the concentration of *Ca*_*cyt*_ at which the reaction rate is half of V_serca_. Although T cells express both SERCA 2b and SERCA 3 isoforms, which have different affinities for Ca^2+^ and maximal pumping rate, we simplified the model by lumping these two isoforms into one average SERCA pump with a unique maximum velocity and Ca^2+^ affinity.

#### Ca^2+^ fluxes through the mitochondria

Ca^2+^ intake in the mitochondria through the uniporter is modeled with a 4^th^ order Hill function [38,39]:

$$J_{mitin}=V_{mitin}∙\frac{{Ca_{cyt}}^{4}}{{Ca_{cyt}}^{4}+{K_{mitin}}^{4}}$$ (eqn 14)

where *V*_*mitin*_ is the maximum rate of Ca^2+^ uptake in the mitochondria and *K*_*mitin*_ is the concentration of *Ca*_*cyt*_ at which the reaction rate is half of *V*_*mitin*._

Ca^2+^ efflux from the mitochondria through the Na^+^/Ca^2+^ exchanger and permeability transition pores (PTP) is given by the lumped expression [31,32]:

$$J_{mitout}=V_{mitout}∙{Ca}_{mit}∙\frac{{Ca_{cyt}}^{2}}{{Ca_{cyt}}^{2}+{K_{mitout}}^{2}}$$ (eqn 15)

where *V*_*mitout*_ is the maximum rate of Ca^2+^ efflux and *K*_*mitout*_ is the concentration of *Ca*_*cyt*_ at which the reaction rate is half of *V*_*mitout*._

#### Ca^2+^ fluxes through the plasma membrane

The details of store operated calcium entry (SOCE) have only been uncovered recently. Previous mathematical descriptions of *J*_*crac*_ include second order Hill dynamics with respect to either IP_3_ levels [32] or cytoplasmic Ca^2+^ levels [40], as well as a phenomenological model involving a diffusible messenger, Ca^2+^ diffusible factor (CIF) [41]. More recently, after the discovery of the STIM1 and ORAI1 proteins and their interaction, Liu *et al*. [42] and Chen *et al*. [43] have attempted to provide a more accurate mathematical description of SOCE, by including activation and dimerization of STIM1, association with the ORAI1 CRAC channels, and CRAC activation. More specifically, Liu *et al*. designed SOCE as a feedback controller that rejects disturbances and tracks Ca^2+^ levels in the cytosol and in the ER [42]. We simplified this system by neglecting the delay formed by STIM1 activation and assuming that the binding of STIM1 to ORAI is at a steady state only depending on the concentration of Ca^2+^ in the ER. Therefore, *J*_*crac*_ can be expressed as:

$$J_{crac}=V_{crac}∙\frac{K_{stim}^{3}}{Ca_{ER}^{3}+K_{stim}^{3}}∙\frac{Ca_{ext}}{Ca_{ext}+K_{soc}}$$ (eqn 16)

where *V*_*crac*_ is the maximum Ca^2+^ influx through the CRAC channels, *K*_*soc*_ is the concentration of *Ca*_*ext*_ at which the half maximal observed reaction rate is achieved and *K*_*stim*_ is the dissociation constant of ER Ca^2+^ to STIM1.

Ca^2+^ influx through the plasma membrane is also permitted through a plasma membrane leak and is given by:

$$J_{PMleak}=K_{PMleak}∙Ca_{ext}$$ (eqn 17)

where *K*_*PMleak*_ is the rate of leakage through the plasma membrane.

Ca^2+^ efflux from the cytosol to the extracellular space is mainly due to the PMCA pumps and is described as:

$$J_{pmca}=V_{pmca}∙\frac{{Ca_{cyt}}^{2}}{{Ca_{cyt}}^{2}+{K_{pmca}}^{2}}$$ (eqn 18)

where *V*_*pmca*_ is the maximal PMCA efflux rate and *K*_*pmca*_ is the concentration of *Ca*_*ext*_ at which the reaction rate is half of *V*_*pmca*._

### Model Optimization and Simulation

The series of differential equations were solved using Matlab R2014b (Mathworks, Natick, MA). The ODE solver for stiff system ode23s was used. Initial conditions were chosen according to published experimental data before parameter optimization or computed at steady state (Table 1).

**Table 1:** Initial conditions for Jurkat Model and Primary CD8^+^ T Cell Models.

State Variable	Jurkat Model Initial Condition	Primary CD8^+^ T Cell Model Initial Condition	Reference
**PLCγ**	70 nM	70 nM	This work
**IP**_**3**_	0.54 μM	0.54 μM	[44]
**Ca**_**cyt**_	50 nM	50 nM	[45]
**Ca**_**ER**_	350 μM	280 μM	350 [45]
**Ca**_**mit**_	0.1 μM	0.1 μM	[46] followed by steady state computation: J_mitin_ = J_mitout_
**h**	0.1	0.1	
**R**	10	10	

Parameter estimation was performed by estimating the difference between the experimental data and the corresponding model prediction (sum of squared error) using a genetic algorithm followed by a combination of Matlab Global Optimization Toolbox functions for constrained nonlinear programing (fmincon) and pattern search algorithm (patternsearch). Since the model parameters were estimated to fit different experimental conditions, the objective function consisted in the sum of errors across experimental conditions for fitting the Jurkat and Young CD8^+^ T Cell Model:

$$S=\sum_{t=1}^{t_{sim}}\sum_{n=1}^{N}\sum_{c=1}^{C}{\left(\frac{x_{pred}(c,n,t)-x_{exp}(c,n,t)}{x_{data}(c,n,t)}\right)}^{2}$$ (eqn 19)

where *t*_*sim*_ is the maximal simulation time, *N* the number of state variables used for optimization and *C* the number of experimental conditions being optimized. The Old CD8^+^ T Cell Model was fit with a similar, slightly altered error function to account for differences in peak amplitude (*peak*_*Amp*_ and *peak*_*ExpAmp*_ for the model and experimental data, respectively):

$$S=\sum_{t=1}^{t_{sim}}\sum_{n=1}^{N}\sum_{c=1}^{C}{\left(\frac{\frac{x_{pred}(c,n,t)}{peak_{Amp}}-\frac{x_{exp}(c,n,t)}{peak_{ExpAmp}}}{\frac{x_{exp}(c,n,t)}{peak_{ExpAmp}}}\right)}^{2}$$ (eqn 20)

The parameter bounds were selected based on previously published experimental or modeling parameter data (Table 2). For fitting experimental data, Ca^2+^ dynamics were experimentally measured following TCR ligation in three different conditions: no inhibitor, 50 μM EGTA or 100 μM TMB-8.

**Table 2:** Model parameters bounds for optimization.

Parameter	Bounds	Source/Explanation
***β*_*i*_**	[0.001 1]	SS value: 0.009 [29]
***β*_*er*_**	[0.001 1]	SS value: 0.196 [29]
***β*_*mit*_**	[0.001 1]	0.0025 [28,29]
***ρ*_*er*_**	0.015	[47]
***ρ*_*mit*_**	0.08	[47]
***k*_*PLCact*_**	[0.001 0.01] s^-1^	0.047 [48]
***k*_*PLCdeact*_**	[0.01 0.1] s^-1^	
***k*_*IP*3*prod*_**	[0.1 1] μM^-1^ s^-1^	1 [48]
***k*_*IP*3*deg*_**	[0.01 0.1] s^-1^	
***V*_*IP*3_**	[0.05 80] s^-1^	0.189 [32], 3 [39], 1.11 [40], 66.6 [43]
***K*_*IP*3_**	[0.1 1] μM	0.136 [32], 0.13 [40], 1 [43], 3 [39]
***K*_*act*_**	[0.05 0.5] μM	0.0814 [32], 0.08 [40], 0.4 [43], 0.13 [39]
***A***	[0.01 0.5]	0.104 [32], 0.032 [40], 0.5 [43]
***K*_*inh*_**	1 μM	1 [32]
***K*_*IP*3*inh*_**	[0.5 1.5] μM	1.05 [32]
***K*_*ERleak*_**	[0.0005 0.05] s^-1^	0.002 [32], 0.02 [40], 0.0009 [43], 0.01 [39], 0.002 [42]
***V*_*serca*_**	[0.2 250] μM s^-1^	114 [32], 0.9 [40], 1 [43], 0.27 [39], 1 [48]
***K*_*serca*_**	[0.15 0.8] μM	0.754 [32], 0.1 [40], 0.15 [43], 0.175 [39], 0.2 [48]
***V*_*mitin*_**	[100 800] μM s^-1^	300 [31], 506 [32]
***K*_*mitin*_**	[0.5 1.5] μM	0.8 [31], 1 [32], 0.6 [39]
***V*_*mitout*_**	[50 500] μM s^-1^	125 [31], 476 [32]
***K*_*mitout*_**	[1 10] μM	5 [31,32]
***V*_*crac*_**	[0.01 10] μM s^-1^	0.226 [32], 8.85 [42], 0.01 [49]
***K*_*soc*_**	[50 1000] μM	500 [42]
***K*_*stim*_**	[150 250] μM	152.3 [50]
***K*_*PMleak*_**	[2.5e-7 3.5e-5] s^-1^	5.6e-6 [49], 2.6e-7 [40], 4.6e-7 [39], 3.3e-5 [32]
***V*_*pmca*_**	[0.01 50] μM s^-1^	0.05 [49], 0.01 [40], 0.013 [39], 0.0893/0.59 [32], 38 [42]
***K*_*pmca*_**	[0.1 0.5] μM	0.12 [40], 0.2 [39], 0.113/0.44 [32], 0.5 [42]
***K*_*STIMpmca*_**	[5 450] μM	Range in ER Ca^2+^ concentration

Sensitivity analysis was performed by perturbing each parameter value (one at a time) by 1 to 20% and comparing the new peak time, amplitude and decay constant to the feature values without perturbation:

$$Sensitivity=\frac{\Delta feature/feature}{\Delta p/p}$$ (eqn 21)

where *p* is the specific parameter used to perform the sensitivity analysis.

### Parameter and species fitting

The computational model for TCR induced Ca^2+^ signaling in lymphocytes was first developed for Ca^2+^ signaling in Jurkats and then adapted for primary CD8^+^ T cells (Table 3). The model consists of 7 state variables and 29 parameters. It is divided into two major submodules. The first one represents TCR stimulation and PLC-γ phosphorylation. The second module corresponds to IP_3_ formation and the downstream cytoplasmic Ca^2+^ increase. These modules were fit to temporal changes in IP_3_ concentration in the presence of EGTA [44] and experimental Ca^2+^ time courses in the absence or presence of the chemical inhibitors EGTA and TMB-8. EGTA is a Ca^2+^ chelator that buffers extracellular Ca^2+^ and will reduce external Ca^2+^ entry through the CRAC channels and PM leakage. TMB-8 is an IP_3_R blocker that will prevent the opening of the IP_3_R channel and therefore limits ER store Ca^2+^ release. To fit the Ca^2+^ time courses obtained from cells treated with inhibitors, two additional parameters were added, λ_1_ and λ_2_ that represent the percent reduction in extracellular Ca^2+^ and in J_IP3_, respectively. λ_1_ is set to 0.33 and λ_2_ was fit to 0.30. The optimization pipeline was repeated multiple times for each of the models and the results are compiled using the *shadedErrorBar* function from MATLAB^®^ Central. For the Jurkat Model, 17 different parameter sets were obtained from the same fitting equation and can be seen in S2 Fig. The average trace is computed across all optimized parameter sets and the standard deviation of this trace is shaded around the average.

**Table 3:** Optimized parameter values for the Jurkat cell model and the Young CD8^+^ T cell model.

Parameter	Jurkat T cells	Primary CD8^+^ T cells
	Mean +/- SD	Mean +/- SD
***β***_i_*	0.043 +/- 0.054	0.017 +/- 0.019
***β***_er_*	0.077 +/- 0.033	0.54 +/- 0.33
***β***_***mit***_	0.21 +/- 0.20	0.033
***ρ***_***er***_	0.015	0.015
***ρ***_***mit***_	0.08	0.08
***k***_***PLCact***_	0.0038 +/- 0.00091 s^-1^	0.0033 s^-1^
***k***_***PLCdeact***_	0.047 +/- 0.019 s^-1^	0.042 s^-1^
***k***_***IP*3*prod***_	0.47 +/- 0.18 μM^-1^ s^-1^	0.48 μM^-1^ s^-1^
***k***_*IP*3deg_*	0.011 +/- 0.0024 s^-1^	0.051 +/- 0.021 s^-1^
***V***_***IP*3**_	3.3 +/- 3.7 s^-1^	4.0 s^-1^
***K***_***IP*3**_	0.37 +/- 0.12 μM	0.57 μM
***K***_***act***_	0.14 +/- 0.042 μM	0.13 μM
***A***	0.090 +/- 0.025	0.079
***K***_***inh***_	1 μM	1 μM
***K***_*IP*3inh_*	0.87 +/- 0.22 μM	1.4 +/- 0.17 μM
***K***_ERleak_*	0.0031 +/- 0.0015 s^-1^	0.034 +/- 0.011 s^-1^
***V***_serca_*	68 +/- 35 μM s^-1^	173 +/- 49 μM s^-1^
***K***_***serca***_	0.37 +/- 0.094 μM	0.43 μM
***V***_***mitin***_	407 +/- 176 μM s^-1^	389 μM s^-1^
***K***_***mitin***_	0.83 +/- 0.18 μM	0.81 μM
***V***_mitout_*	166 +/- 50 μM s^-1^	295 +/- 132 μM s^-1^
***K***_mitout_*	4.06 +/- 1.7 μM	5.4 +/- 2.1 μM
***V***_***crac***_	1.3 +/- 0.42 μM s^-1^	2.4 μM s^-1^
***K***_soc_*	366 +/- 160 μM	326 +/- 233 μM
***K***_***stim***_	215 +/- 28 μM	178 μM
***K***_***PMleak***_	2.8e-5 +/- 1.2e-5 s^-1^	1.1e-6 s^-1^
***V***_pmca_*	1.6 +/- 0.73 μM s^-1^	0.52 +/- 0.78 μM s^-1^
***K***_***pmca***_	0.18 +/- 0.090 μM	0.11 μM

For parameters that were fit to experimental data, the mean +/- SD is given as the culmination of 17 Jurkat optimization runs and 15 Primary CD8^+^ T Cell optimization runs. Parameters were varied within the original bounds for those marked with an asterisk.

Our computational model of TCR-induced Ca^2+^ signaling in Jurkats was then adapted to describe Ca^2+^ signaling in low passage primary CD8^+^ T cells. The model was optimized to fit Ca^2+^ time courses from low passage primary CD8^+^ T cells while keeping many parameters conserved between both cell types and allowing the starred species in Table 3 to vary within the original bounds. For the Young CD8^+^ T cell model, parameter estimation was performed with a genetic algorithm in Matlab R2014b (Mathworks, Natick, MA). The initial parameter set was populated from the best parameter fit +/- 20% of the Jurkat Ca^2+^ model. The model was fit to conditions without inhibitors and was validated by predicting Ca^2+^ dynamics in the presence of chemical inhibitors. Similar to the Jurkat Model fitting, 15 runs were completed of this optimization algorithm and the results are compiled using the *shadedErrorBar* function from MATLAB^®^ Central.

## Results

### Ca^2+^ signaling of *ex vivo* aged T cells

**Fig 1:**
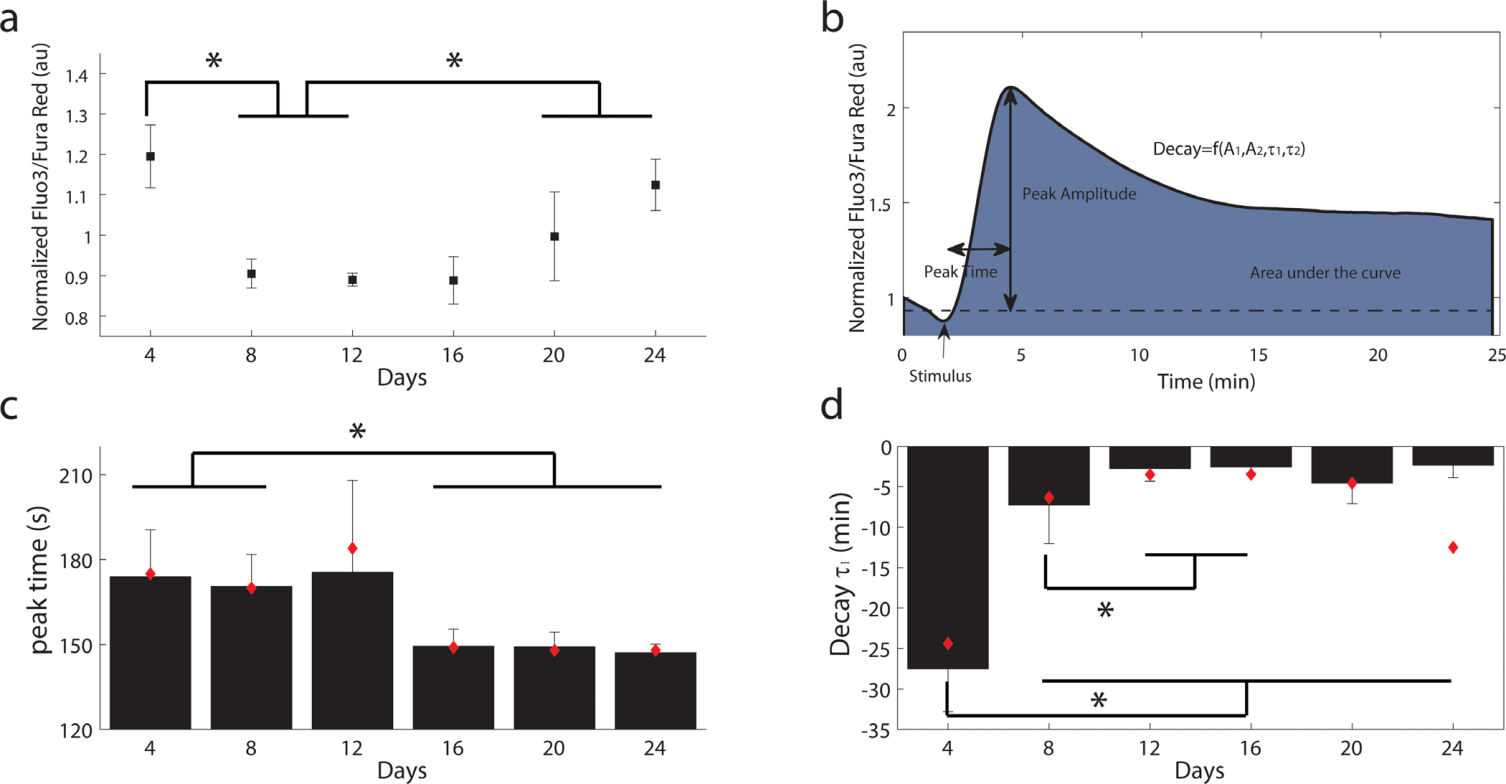
Age related Ca^2+^ changes in CD8^+^ T cells. a) Cytoplasmic Ca^2+^ levels in resting T cells. Mean and SEM from 4 different donors. AU = arbitrary units b) Representative trace of Ca^2+^ dynamics following TCR stimulation with the related parameters studied, peak time, peak amplitude, baseline and decay. The integral corresponds to the colored area under the curve. c) Time to peak in seconds. d) Fast decay time constant in minutes. For c-d), the data represents the mean of each calculated parameters for each donor and its standard deviation. The red diamonds correspond to the parameter calculated if the Ca^2+^ time courses are averaged for all donors for a specific day in culture.

Using a long-term culturing protocol to accelerate cell divisions of human primary CD8^+^ T cells [7], we measured baseline cytosolic Ca^2+^ and dynamic responses to TCR activation by flow cytometry. In previous work with this culturing procedure, we established that immunosenescence, as defined by an inability to divide, is achieved within 12 population doublings in approximately 24 days in culture [7]. In CD8^+^ T cells, we observed elevated resting levels of cytoplasmic Ca^2+^ in young cells after only a few days in culture (days 5–6) and old cells after a prolonged time in culture (days 20–24) when the cells had plateaued in division rate (Fig 1A). In both young and old cells, T cell receptor activation resulted in dynamic changes in cytosolic Ca^2+^ levels. To quantitatively define the differences observed among the dynamic traces, we defined the following parameters from a representative Ca^2+^ time course: peak amplitude, peak time, area under the curve as well as four additional parameters to describe the decay due to the SERCA and CRAC channel opening, coefficients A_1_ and A_2_, and decay constants τ_1_ and τ_2_ (Fig 1B, Eq 1). An example of an individual donor calcium response for young and old cells can be found in S1 Fig. From these 7 parameters, only the peak time and the decay constant τ_1_ showed statistically significant differences with time in culture (p<0.05). Older T cells reached their peak amplitude faster and had a faster decay time constant (Fig 1C and 1D). We did not observe monotonic trends in peak amplitude, integral or the second decay time constant.

### Computational modeling of Ca^2+^ signaling

**Fig 2:**
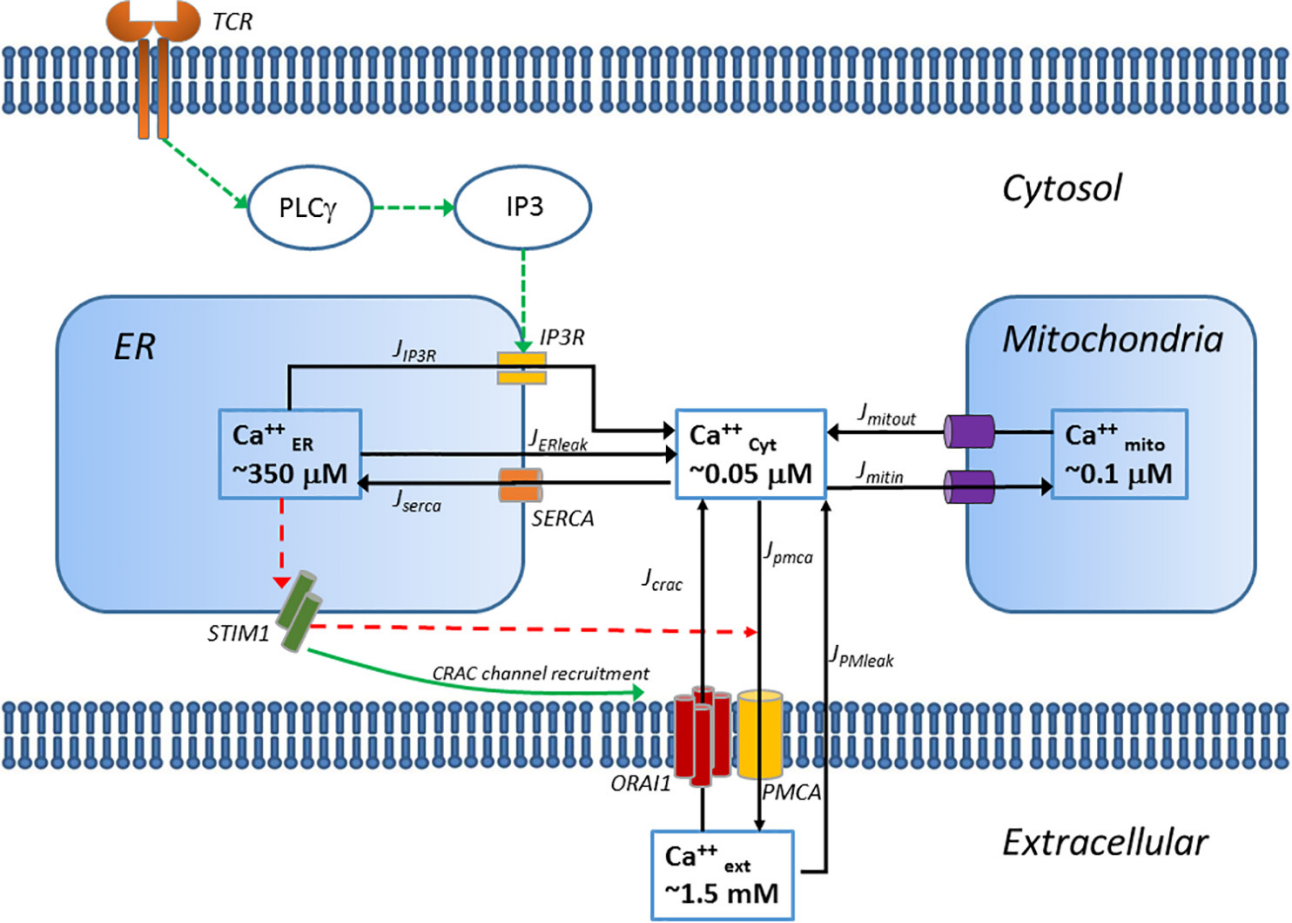
Schematic of the Ca^2+^ signaling model.

We then developed a computational model for calcium signaling after T cell receptor ligation to investigate the differences in the calcium signaling network between the young and old primary CD8^+^ T cells. The model is comprised of a simplified module for IP_3_ formation and calcium fluxes from the three major cellular compartments: cytosol, endoplasmic reticulum (ER), mitochondria as well as the extracellular space as shown in Fig 2. This model is based on previously published models of calcium dynamics [31,32,39,42] with additional terms reflecting the more recently discovered stromal interaction molecule STIM1 and CRAC subunit ORAI1.

**Fig 3:**
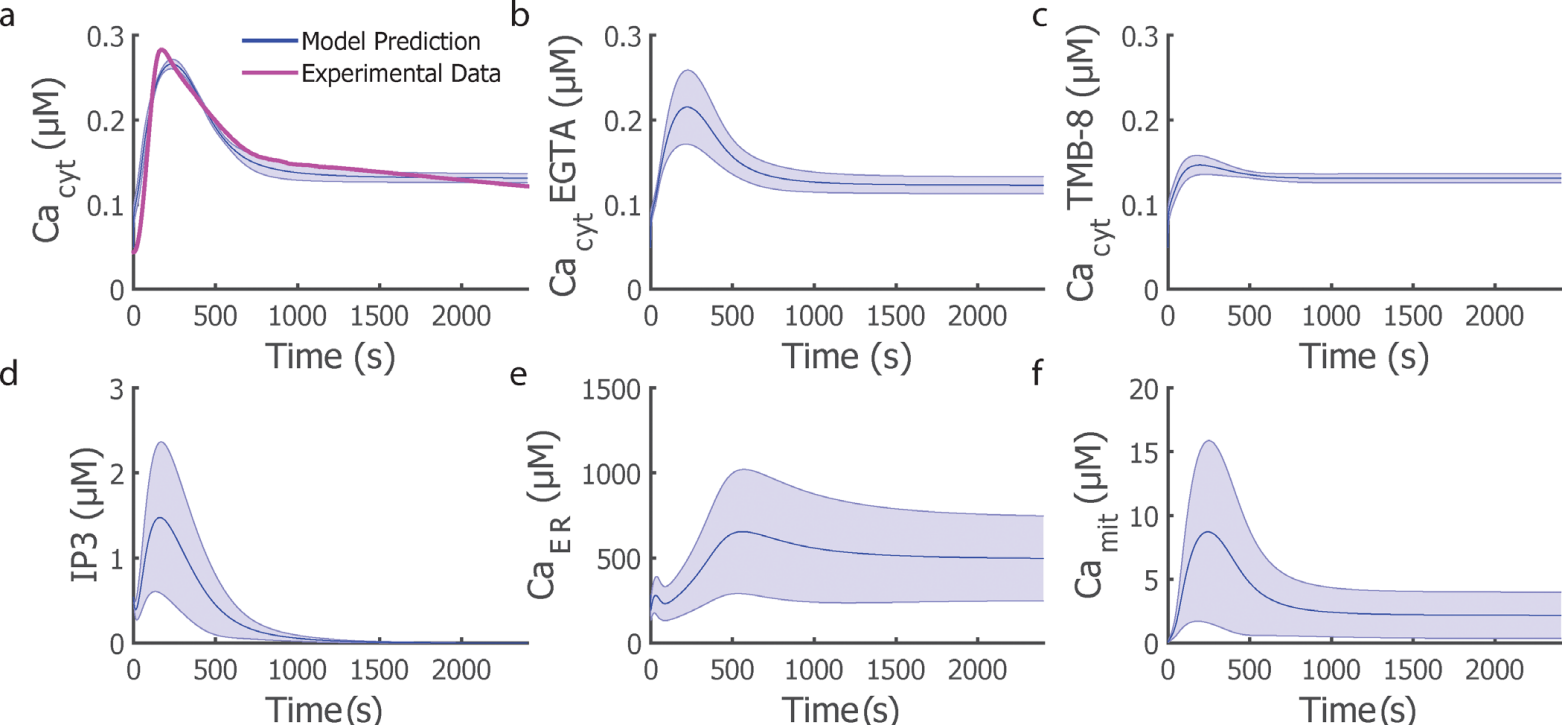
Young CD8^+^ T Cell Model predictions of IP_3_ and Ca^2+^ in the various cellular compartments in response to TCR signaling in CD8^+^ T cells. Cytosolic Ca^2+^ dynamics in the absence (a) or presence of inhibitors (b,c) at the same concentration as for Jurkat cells. d-f) model predictions for other state variables in the no inhibitor simulation. The average trace across all optimized parameter sets is illustrated by the blue solid line and the shaded region represents the standard deviation of the dynamic behavior.

To optimize and validate our computational model of Ca^2+^ signaling in T cell activation, we optimized the model to fit Ca^2+^ signaling dynamics in activated Jurkat cells for a total of 17 different parameter sets (S2 Fig) and then optimized the model for Ca^2+^ signaling in young CD8^+^ T cells by fitting our model to cytosolic Ca^2+^ dynamics from primary cells expanded to days 5–6 and using initial values obtained from the Jurkat cell model (S3 Fig & S4 Table). The young primary CD8^+^ T cell Ca^2+^ signaling model was used to predict IP_3_ and Ca^2+^ in the cytosol, mitochondria and ER as shown in Fig 3. Because the time scales of Ca^2+^ dynamics are quite different in the Jurkat CD4^+^ T cell line and primary CD8^+^ T cells (rise time of 30 sec versus 150 sec), the optimized parameter values are quite different for those two cell types, especially the maximum velocities (Table 3).

### Changes in mRNA levels of plasma membrane channels are not sufficient to explain changes in cytoplasmic Ca^2+^ dynamics due to long-term culture

**Fig 4:**
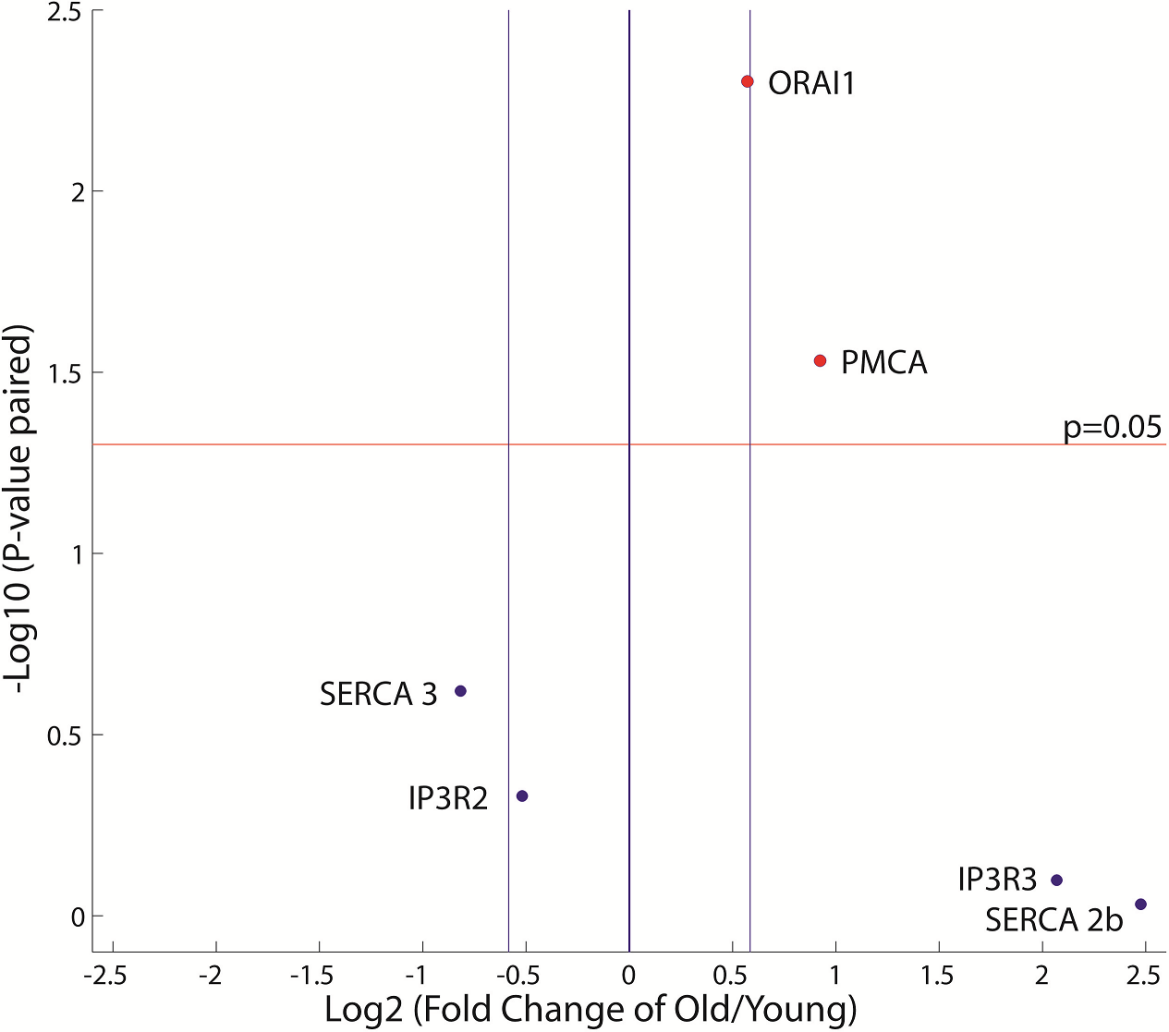
Changes in mRNA levels of Ca^2+^ channels and pumps with age (n = 6). Points above the red line represent targets that show significant statistical difference (at p < 0.05) between young and old samples. The blue lines represent fold changes that are above 1.5 fold up or down.

To determine if changes in Ca^2+^ signaling dynamics are due to changes in expression of the proteins involved in the Ca^2+^ signaling pathway, we measured mRNA levels of the major Ca^2+^ channels and pumps expressed in T cells (IP3R2, IP3R3, SERCA2B, SERCA3, ORAI1 and PMCA) for young (days 4–8 in culture) and old cells (days 20–24 in culture). Out of these six targets, PMCA and ORAI1 showed significant upregulation (p < 0.05) with age (Fig 4). We then investigated whether implementing new initial conditions of PMCA and CRAC channels is sufficient to explain the faster peak time and decay observed during aging. The absolute number of channel/pump proteins would directly affect the maximum flux through these proteins; hence we used the model described above and let the parameters *V*_*crac*_, *V*_*pmca*_ vary in the original specified bounds to fit data from old cells (day 20–24 in culture). These two parameters alone were not sufficient to fit simultaneously the time-to-peak, decay time constant, and amplitude (S5 Fig presents the best fit in respect to amplitude, time-to-peak and decay time constant). The model therefore suggests that changes at the protein abundance level among the calcium sequestration/release regulators are not solely responsible for the changes in calcium dynamics observed as a function of change.

### Model Predictions

**Fig 5:**
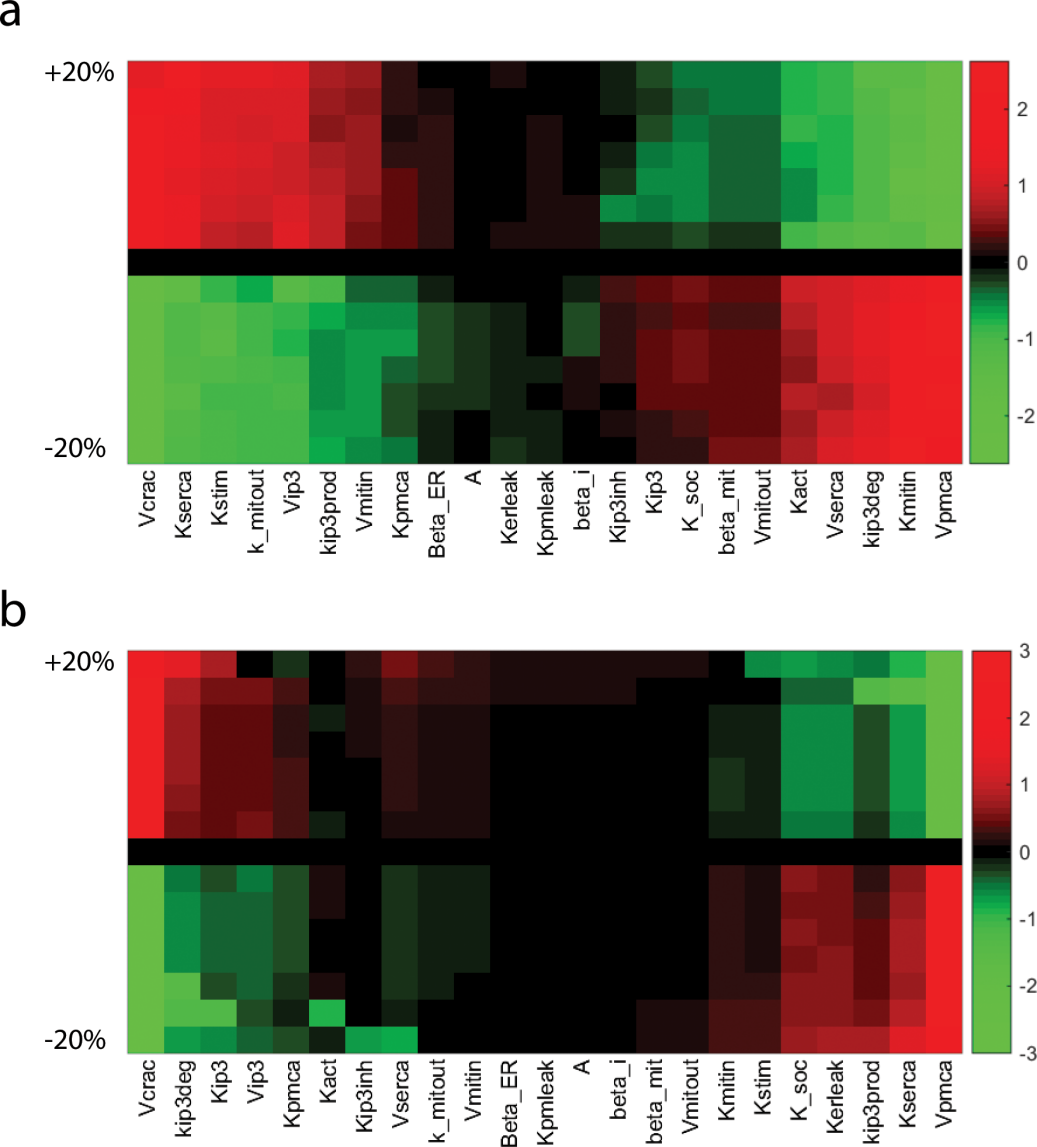
Model sensitivity analysis of immunosenescent features. a) Parameter sensitivity to the feature peak time. b) Parameter sensitivity to the feature decay time constant τ_1_. Parameters values were perturbed by a percentage up to 20% up and down and were clustered for easier visualization.

Because changes in the levels of PMCA and CRAC channels were insufficient to explain the changes in Ca^2+^ dynamics with time in culture, we identified which parameters were most responsible for the time-to-peak and decay time constant (the two metrics with statistically significant differences with days in culture in Fig 1) by performing a sensitivity analysis on the Young CD8^+^ T Cell model (Fig 5, S4 Fig, and S5 Table). Each parameter was perturbed individually, and the features (peak time and decay time constant) were measured for the new model output. For these two features, several parameters exhibit nonlinear behaviors; for instance certain parameter combinations led to oscillatory behaviors which might affect the calculated features. Higher parameter sensitivity to the feature decay time than peak time can be observed; however, the parameters involved in altering both the peak time and decay time constant are fairly consistent. Amongst the initial 24 parameters tested, seven parameters were identified as being the main drivers of the observed changes with age, many of which are involved with Ca^2+^ exchange with the ER stores (Fig 5). The seven parameters found to have the most effect on peak time and the decay constant were *K*_*serca*_, *V*_*pmca*_, *V*_*crac*_, *K*_*stim*_, *K*_*IP*3_, *K*_*IP*3*prod*_, and *K*_*IP*3*deg*_ (Fig 5).

**Fig 6:**
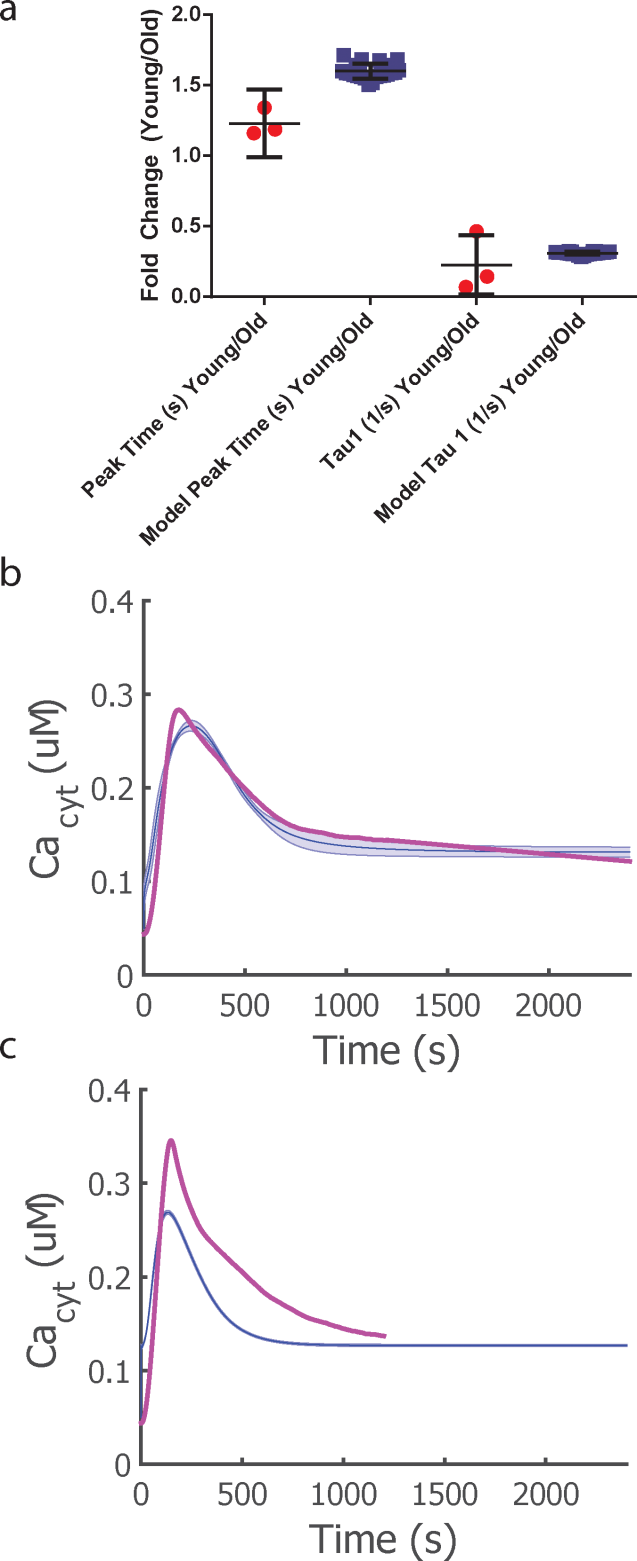
Model predictions of Ca^2+^ dynamics in old CD8^+^ T cells. a) Comparison of experimental data to the model predictions for the two identified parameters, Peak Time and Decay Constant τ_1_. Error bars represent mean and 95% confidence interval represented in the above error bar. b) Young cytosolic Ca^2+^ model trace (blue) compared to experimental data (pink). c) Old cytosolic Ca^2+^ model trace (blue) compared to experimental data (pink). The average trace across all optimized parameter sets is illustrated by the blue solid line and the shaded region represents the standard deviation of the dynamic behavior.

To ensure these parameters were the drivers of the observed old T cell phenotype, we used the parameter set from the Young CD8^+^ T cell model and simultaneously varied these seven parameters to fit the old T cell time course using a genetic algorithm approach. This process was repeated 24 times to achieve 24 different parameter sets for comparison to the Young CD8^+^ T Cell Model. The objective function consisted of the sum of squared difference between the model and the experimental time course (Eq 20), with additional constraints for peak time and peak amplitude. Fig 6C and S6 Fig presents the best Old CD8^+^ T Cell Model fit. The new optimized parameter set shows differences compared to the Young CD8^+^ T cell model best parameter set, as shown in Table 4 as calculated with ((n_Young_-n_Old_)/n_Young_)*100%, where n_Young_ is the parameter value in the Young CD8^+^ T cell Model and n_Old_ is the parameter value in the Old CD8+ T Cell Model.

**Table 4:** Difference in fit parameter values between the Young and Old CD8^+^ T Cell Models.

Parameter	Percentage Change +/- SD (%)
***K***_***serca***_	-2.59 +/- 5.80
***V***_***pmca***_	-27.3 +/- 35.2
***V***_***crac***_	-53.1 +/- 39.3
***K***_***stim***_	2.18 +/- 11.6
***K***_***IP*3**_	79.8 +/- 4.54
***K***_***IP*3*prod***_	-16.6 +/- 5.90
***K***_***IP*3*deg***_	-433 +/- 34.0

Because we typically associated changes in Vmax with altered protein abundance and changes in binding rate constants with intrinsic changes to proteins (i.e. post-translational modifications), this motivated our investigation of redox metabolic reprogramming during the *ex vivo* aging protocol that may induce oxidative post-transcriptional changes in SERCA, IP3, and STIM1 and correspondingly alter kinetic parameters. In S7 and S8 Figs, we varied the two main parameters associated with STIM1, *V*_*crac*_ and *K*_*stim*_, individually to determine the effect on the calcium traces. We found that both of these parameters alone altered the peak time and decay of the calcium signaling when varied +/- 20% of the optimized Young CD8^+^ T cell model, which supports our model predictions that STIM1 may be involved with age related changes in T cells.

### Age-related modifications in overall cellular redox status

Gene expression profiles of 84 antioxidant and redox related genes (S1 Table) were measured from young (up to 8 days in culture, corresponding to a maximum of 3 population doublings) and old (above 15 days in culture, corresponding to more than 8 population doublings) CD8^+^ T cells of healthy adult donors. Out of these 84 genes, only 58 were expressed in T cells (S1 Table). The inter-array coefficient of variation (CV) represents the donor to donor variability in gene expression and ranged from 0.9% to 19.06%, with a mean percentage of 4.4%. The list of all targets, their corresponding fold changes and p-values can be found in S2 Table. Significant changes associated with time in culture (p<0.05) were observed in 6 genes; notably, Dual oxidase 1 (Duox1) and Glutathione peroxidase 3 (Gpx3) were upregulated during *ex vivo* expansion. Among downregulated genes in older T cells (p<0.1) were Glutaredoxin 2 (Glrx2), and Thioredoxin reductase 1 (Txnrd1). To validate the PCR array results and compare mRNA levels to protein expression, we measured the relative levels of the H_2_O_2_-producing enzyme Duox1 by Western blot analysis, which confirmed increased levels of Duox1 (S7 Fig).

**Fig 7:**
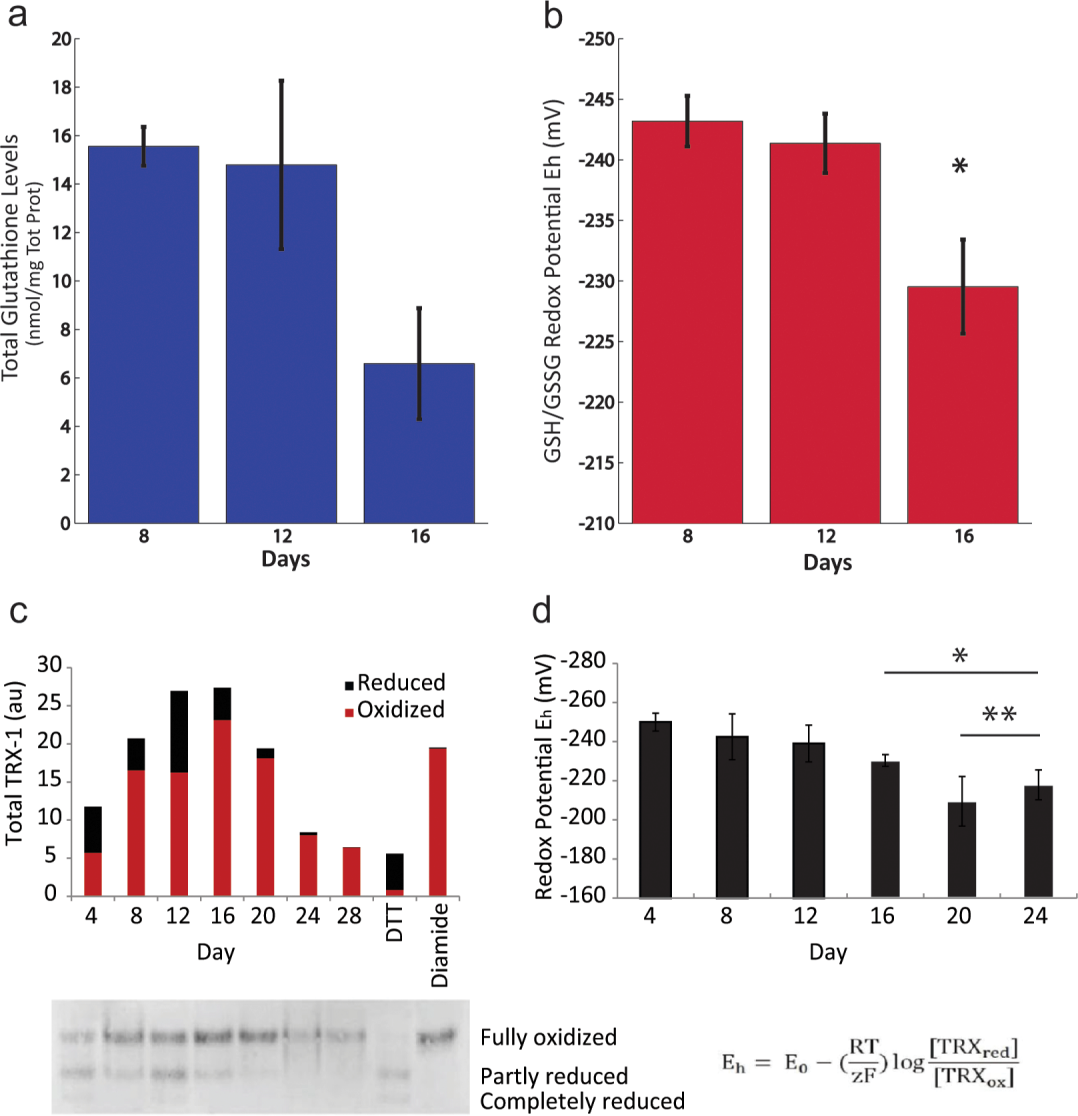
An oxidative shift in redox potential with ex vivo T cell expansion. Total glutathione levels (a) and corresponding GSH/GSSG redox potential (b) in CD8^+^ T cells with time in culture (n = 3). Statistical analysis: one-way ANOVA (p = 0.04) followed by Scheffe’s post-hoc test * p<0.05 between day 8 and day 16. Trx1 levels in CD8^+^ T cells with time in culture. c) Total, reduced and oxidized Trx1 levels for a representative donor. DTT-treated lysate is included as a reduced control and diamide-treated lysate is included as an oxidized control. d) Trx1 redox potential (n = 4). Statistical analysis: one-way Anova (p = 0.007) followed by Scheffe’s post-hoc test * p<0.05 between day 4 and days 16–24. ** p<0.05 between days 8–12 and days 20–24.

We investigated whether these age-related changes in gene expression corresponded to changes in two cellular redox couples, including the glutathione thiol/disulfide (GSH/GSSG) and thioredoxin 1 (Trx-1) redox couples, as measures of cellular oxidation. The glutathione thiol/disulfide redox couple (GSH/GSSG) is the predominant mechanism for maintaining the intracellular microenvironment in a highly reduced state that is essential for antioxidant/detoxification capacity, redox enzyme regulation, transcription of antioxidant response elements (ARE) and adequate immune response. Total levels of glutathione did not change significantly with time in culture (p = 0.13); however, the ratio of oxidized to reduced glutathione did increase, as indicated by changes in the GSH/GSSG redox potential (Fig 7A and 7B). The thioredoxin reductive system is also an essential cellular mechanism facilitating the reduction of ROS by supporting the peroxidase action of peroxiredoxins and reducing protein disulfides. Pooled total levels of Trx-1 did not show significant changes among our four donors (data not shown); yet at the individual donor level, total Trx-1 expression was reduced at longer time in culture compared to day 4 and 8 (Fig 7C). The proportion of oxidized Trx1 increased with time in culture, leading to an overall increase in the cellular Trx1 redox potential (Fig 7D).

### Redox regulation of STIM1 changes with aging

**Fig 8:**
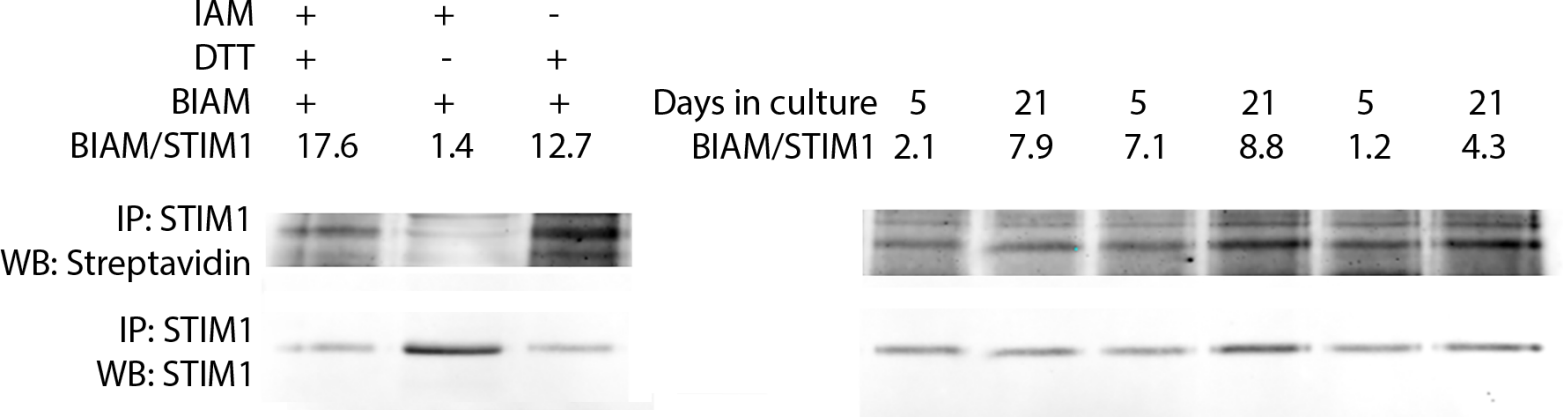
Oxidation of STIM1 thiols occurs during ex vivo aging of primary T cells. a) Validation of assay for detection of oxidation (streptavidin signal) of STIM1 in Jurkat lysates. Reduced thiols were labelled with PEO-biotin-iodoacetamide (BIAM) as described in the Methods. b) Oxidation of STIM1 in young and old primary human CD8^+^ T cells.

Based on our model’s predictions of STIM1-mediated PMCA inhibition as a key factor in describing Ca^2+^ signaling dynamics in older T cells, our findings that T cells shift towards a more oxidizing intracellular environment with days in culture, and literature reports of STIM1 as a redox sensor [21], we hypothesized that STIM1 oxidation changes with the *ex vivo* expansion protocol. We developed an assay to detect changes in cysteine redox regulation of STIM1 by measuring reversibly oxidized cysteines including sulfenic acids, S-glutathionylation, and disulfides. By protecting reduced cysteines via alkylation during cell lysis and then reducing reversibly oxidized cysteines after STIM1 immunoprecipitation, we quantified cysteine oxidation of STIM1 via biotinylation of these nascent thiols with PEO-biotin-iodoacetamide (BIAM). Jurkat cell lysates were used as assay controls as shown in Fig 8 to confirm that the thiol chemistry worked and to demonstrate the assay discriminates between reduced and oxidized thiols. For example, Jurkat lysates were treated with iodoacetamide but not DTT to validate that thiols in the reduced state in the cell were fully alkylated. The low streptavidin signal confirmed that fewer cysteines were available for biotinylation than samples treated with DTT. In contrast, Jurkat lysates treated with DTT but without iodoacetamide had more cysteines available for biotinylation. By normalizing the streptavidin signal to the STIM1 signal, we demonstrated a 10-fold increase in signal when comparing oxidized to reduced controls (Fig 8).

Primary CD8^+^ T cells from three different donors were subjected to the accelerated cell divisions *ex vivo* and changes in redox regulation of STIM1 were quantified using this assay. The streptavidin signal was normalized to the STIM1 signal across each sample. As shown in Fig 8, the ratio of oxidized cysteines to STIM1 increased with prolonged culture time across all three donors. In parallel, we tested whether STIM1 expression changed, and we found that STIM1 expression did not change in these same samples (S10 Fig). Our results indicate that basal STIM1 cysteine oxidation increases with the number of population doublings *ex vivo*.

## Discussion

Although the exact causes of functional decline of T cells with age are not known, several studies have demonstrated the development of defects in the early signal transduction events inducing Ca^2+^ release with immunosenescence [6,7]. Altered Ca^2+^ dynamics in T cells have been associated with several age-related diseases, such as neurodegenerative, autoimmune and inflammatory disorders [51]. Because the dynamics of Ca^2+^ signaling are a marker of T cell function in response to stimuli and are known to affect downstream cellular response, we asked whether Ca^2+^ signaling dynamics change after a cell population has undergone multiple population doublings. In our culture model, we did not observe a clear trend in baseline Ca^2+^ levels, or a reduction in peak amplitude or sustained levels after stimulation (Fig 1). We hypothesize that the high Ca^2+^ level that we observe at day 4 is a consequence of the high Ca^2+^ levels required for proliferation, and the elevated levels towards the end of our long-term culture towards cellular senescence. More importantly, we observed altered Ca^2+^signaling dynamics after T cell receptor stimulation in older CD8^+^ T cells as evidenced by a faster decay rate and a time to peak 20 seconds faster than younger cells (Fig 1).

To find an underlying mechanism for these dynamic differences, we measured mRNA levels of the main channels and pumps involved in Ca^2+^ handling in T cells and found a small but significant overexpression of the plasma membrane ORAI1 channel and PMCA4b pump in older T cells while expression of the IP_3_R and SERCA isoforms remained unchanged (Fig 4). There are very few studies concerning transcript levels of Ca^2+^ channels during aging, and to our knowledge none performed on lymphocytes. Zaidi *et al*. observed a general loss of PMCA and reduction of PMCA activity from the membrane of murine brain synaptic membranes [52]. Another study reported reduced expression levels of STIM1 and ORAI in muscle fibers isolated from aged mice [53]. Levels of SERCA2b have been shown to stay constant in old rat thoracic aortas [54], while levels of SERCA3 mRNA decreased in old rat central neurons but without a corresponding decrease in the SERCA3 protein levels [55]. Aging was accompanied by a significant increase in the mRNA levels of IP_3_R1 in a rat’s heart [56]. These differences from our findings might be a result of using excitable cells and various animal models.

Intuitively, if the activities of Ca^2+^ channels and pumps in T cells are reduced with age, as it occurs in other cell types [52], simultaneous overexpression of the Ca^2+^ influx and efflux mechanisms from the plasma membrane may be a compensatory way for the older cells to sustain high levels of calcium for downstream signaling. Based upon the current knowledge of molecular mechanisms of Ca^2+^ signaling, this is an unlikely molecular basis for the faster time to peak and decay time constant. To gain a better understanding of the Ca^2+^ signaling pathway and the relative contribution of each flux towards an integrated dynamic cell response, we built a deterministic computational model of Ca^2+^ signaling in T cells after TCR stimulation. Single cell analysis of Ca^2+^ signaling in T cells show a variety of Ca^2+^ signals ranging from infrequent spikes to sustained oscillations and plateaus [45,57]. Because lymphocyte Ca^2+^ oscillations are not synchronized, we have chosen to model Ca^2+^ dynamics from a population rather than the dynamics of a single T cell.

Because of the importance and complexity of Ca^2+^ signaling in various cellular systems, substantial efforts have been devoted to modeling Ca^2+^ dynamics in neurons [58–60], cardiomyocytes and muscle cells [61–66]. Deterministic models of Ca^2+^ kinetics after T cell receptor engagement in Jurkat and murine T cells [48,49] are able to reproduce the rise in cytoplasmic Ca^2+^ after T cell stimulation but do not include extracellular space, mitochondrial buffering and mechanistic details of SOCE. A more detailed computational model of Ca^2+^ dynamics in immune cells has been reported by Maurya *et al*., which predicts temporal responses of Ca^2+^ concentrations for various doses of stimulus and network perturbations in RAW 264.7 macrophages [32,67]. This prior model provided the foundation which we added enhanced description of store-operated calcium efflux.

Using parameter sets from previously published models of Ca^2+^ dynamics did not recapitulate experimental time courses, which is not surprising as parameter values were collected across various cell types and *ex vivo* conditions. To fit the parameters, we took an approach similar to Maurya *et al*.[32]. For each flux, we surveyed the literature for a mathematical formulation, parameter values, and *in vitro* experimental data, which allowed us to specify upper and lower parameter bounds. It is interesting that there is a large discrepancy in legacy values among similar parameters that can be estimated to be three orders of magnitude different (Table 2). The experimental dataset used to optimize the model summarizes the main molecular mechanisms of the Ca^2+^ signaling pathway after TCR stimulation, with the TMB-8 inhibitor condition emphasizing the early ER Ca^2+^ store release and the EGTA inhibitor condition the importance of extracellular Ca^2+^ to sustain elevated Ca^2+^ levels after ER stores have been emptied. If optimized using only the no inhibitor condition, the model will tend to fit the cytosolic Ca^2+^ time course by adjusting the rates of influx and efflux at the plasma membrane; however this set of parameters does not reproduce experimental data acquired under inhibitor conditions. By fitting the model with three experimental conditions, we achieve sets of parameters that recapitulate Ca^2+^ under all experimental conditions. Confidence in our parameters would be improved with additional experimental resolution, for instance Ca^2+^ time courses from cell organelles.

Experimental data were acquired on Jurkat cells, a model CD4^+^ T cell line easy to manipulate; therefore we initially created a “Jurkat cell” model. This model and its parameter values were used as a starting point to create the Young CD8^+^ T cell model. Parameter values between both cell types show significant differences (Table 3), reflecting the differences in the Ca^2+^ time courses between those two cell types. These differences in time scale might be due to differential protein expression (S1–S3 Tables, [68]) and are reflected by large variation in the maximal velocity parameters between these two cell types.

For both cell types, the model is able to capture the fast initial rise and sustained elevated levels of cytosolic Ca^2+^. Interestingly, the model predicted a slow replenishment of the ER stores, and a fast Ca^2+^ buffering by the mitochondria, mirroring Ca^2+^ dynamics in the cytosol. Although the model does not include any spatial components and any additional control feedback, the network structure combined with optimized parameters under different inhibition conditions recapitulates the role of the mitochondria at the ER/mitochondrial junctions [69] and SOCE-dependent Ca^2+^ release via IP3R/RyR while the stores are being replenished [70]. The experimental mRNA data showed both PMCA and ORAI1 were upregulated with age (Fig 4). Because changes in mRNA levels might not translate directly into the same fold changes in the maximal velocities, we varied those parameters in an attempt to capture Ca^2+^ dynamics in aged cells but the best fit was not able to accurately reproduce these dynamics (S5 Fig). Sensitivity analysis of the model identified perturbations in *K*_*serca*_, *V*_*pmca*_, *V*_*crac*_, *K*_*stim*_, *K*_*IP*3_, *K*_*IP*3*prod*_, *K*_*IP*3*deg*_ as best candidates of age-related alterations (Fig 5). All seven selected parameters that were allowed to vary and showed changes between the young and old models, indicating their importance in recapitulating the dynamic information of aging cells.

Because changes in the cellular redox environment are associated with aging, and calcium signaling is redox-regulated; we investigated the expression of redox regulatory genes and two cellular redox couples in our *ex vivo* model. Comprehensive microarray studies have been conducted to compare gene expression profiles in T cells between young and old human subjects [4,16]. These studies report the differential expression of several key redox regulatory genes associated with oxidative stress. Age-dependent increases in the levels of lipid peroxidation and protein oxidation and declines in glutathione levels and activities of antioxidant enzymes in mixed human T cell populations have also been reported[17]. These prior studies indicate an oxidative shift in redox potential *in vivo* as a function of organism age. Despite reports of oxidative DNA damage, such changes have not been characterized for *ex vivo* aging models, and in particular in culture conditions that mimic expansion methods for adoptive cell therapy. Our results indicate that *ex vivo* culture conditions that drive T cells to replicative senescence mimic observed pro-oxidative features of organismal age-induced cellular redox changes in order to alter calcium dynamics. These culture conditions offer a unique advantage of paired comparisons between genetically identical cells over time and thus eliminating confounding factors that lead to great donor-to-donor variability in phenotypic responses. A disadvantage of our experimental design is that the constant stimulation by antibody coated beads may lead to TCR downregulation from the surface of the T cells. Exosome shedding or transcriptional changes in receptor number may partially account for changes in Ca^2+^ signaling and were not monitored in this study. Under identical culture conditions in a prior study, we did quantify CD28 and CD27 surface expression decreasing ~ 50-fold and 3-fold respectively over a 3 week culture time [7]. Our observation of faster responses and no change in Ca^2+^ amplitude in in older cells is therefore surprising if fewer receptors are being engaged.

The shift towards a more oxidizing cellular redox environment we observed in older T cells, can lead to changes in protein cysteine oxidation, a post-translational modification that has been shown to modulate protein activity as well as protein-protein interactions [71]. There is evidence that sulphydryl oxidation is a mechanism of redox regulation of Ca^2+^ signaling [72]. Orai1, PMCA, STIM1 and IP_3_R contain several amino acid residues that are highly susceptible to oxidation [20,21,73,74]. IP_3_R function has been reported to be affected by ROS by increasing IP_3_R sensitivity to cytosolic IP_3_ levels [75] and inducing conformational change on the luminal side leading to modified channel activity [76]. Recombinant human STIM1 has been shown to be glutathionylated, and S-glutathionylation has been demonstrated at Cys-56 in overexpressed STIM1 [21]. To the best of our knowledge, this is the first investigation of endogenous STIM1 oxidation occurring due to oxidative shifts in redox metabolism. In this study, the increase of cysteine oxidation of STIM1 with time in culture reveals redox regulation of STIM1 as a possible molecular mechanism that could explain the decreased PMCA inhibition by STIM1 predicted by our model.

Additional experimental studies need to be performed to measure the overall implications of redox status in *ex vivo* expanded T cells. Single cell analysis has not been the focus of this study; however tools are now amenable for collecting this information [57,77,78] and coupling single cell calcium measurements to dynamic features of oxidation. In particular, the stimulatory conditions used in this study (2 μg/ml antiCD3) and weak antigenic peptide have been reported to induce Ca^2+^ oscillations at the single cell level [79,80] that are not visualized by our flow cytometry data acquisition. As intracellular Ca^2+^ signaling patterns reflect the differentiation status of human T cells [81], a better discrimination between young and old T cells could be achieved by quantifying the heterogeneity of Ca^2+^ signaling patterns in young versus old cells and incorporating these features into our model of Ca^2+^ signaling.

## Conclusion

Altered Ca^2+^ signaling is a hallmark of aging and other various disease states, yet the biomolecular mechanisms leading to these alterations are unknown. To guide new experimental studies, we constructed a computational model of Ca^2+^ signaling in T cells that recapitulates key features of a typical Ca^2+^ time course in both a T cell line and primary T cells. The model predicted protein targets of regulation, such as STIM1, that was altered during *ex vivo* expansion. We observed enhanced oxidation of the cellular environment and direct evidence of basal STIM1 thiol oxidation associated with the ex vivo, long-term culturing protocol.

## Supporting Information

S1 Fig Example flow cytometry median calcium traces from a donor obtained at Day 4 and Day 24. After cold-binding with anti-CD3 and anti-CD28 as described in Materials and Methods, cells were sampled for 3 minutes by flow cytometry before addition of anti-mouse IgG. The average of the fluorescence ratio was calculated at 4 second intervals and then normalized to the average baseline value to provide a fold-change value.
(PDF)

S2 Fig Optimization of Jurkat T Cell Model using data obtained by no inhibitor, TMB-8, and EGTA conditions. Plots represent 17 different optimized parameter sets that were obtained from comparing the model prediction to experimental data.
(PDF)

S3 Fig Jurkat T Cell model behavior by parameter set used to fit Young CD8+ model. (PDF)

S4 Fig Optimized Young CD8^+^ T Cell Model. (PDF)

S5 Fig Best fit of Old CD8^+^ T cell model varying only two parameters, *V*_*crac*_ and *V*_*pmca*_. (PDF)

S6 Fig Best fit of Old CD8^+^ T cell model varying the seven parameters as identified in the sensitivity analysis of the Young CD8^+^ T cell model. (PDF)

S7 Fig Varying *K*_*stim*_ from the Young CD8^+^ T cell model fit to investigate the effects on calcium traces. *K*_*stim*_ was varied +/- 20% the fit value of 178.
(PDF)

S8 Fig Varying *V*_*crac*_ from the Young CD8^+^ T cell model fit to investigate the effects on calcium traces. *V*_*crac*_ was varied +/- 20% the fit value of 2.37.
(PDF)

S9 Fig Validation of RT-PCR results with Duox 1 expression. a) Representative Western Blot. b) Quantification of the Western Blots. Protein levels are normalized to the young cells protein expression level. * p<0.05 (paired 2-tail t-test).
(PDF)

S10 Fig Expression of STIM1 in young and old primary human CD8+ T cells. (PDF)

S1 Table List of all oxidative stress and antioxidant PCR primer targets on the PCR array. Red genes represent targets that are not expressed in CD8+ T cells.
(PDF)

S2 Table Exhaustive list of fold changes and their corresponding p-values in targets expressed in CD8+ T cells. A fold change below 1 corresponds to a downregulation (2^-ΔCT^).
(PDF)

S3 Table Normalized mRNA levels of individual genes expressed in young CD8+ T cells, ranked in descending order of expression (n = 6). (PDF)

S4 Table Optimized parameter set obtained from the Jurkat T Cell Model fitting used for the seeding the initial population of parameter values for the genetic algorithm optimization of the Young CD8+ T Cell Model to experimental data. (PDF)

S5 Table Optimized parameter set obtained from fitting the Young CD8+ T Cell Model to experimental data. This parameter set was used for all sensitivity analysis performed on the Young CD8+ T Cell Model.
(PDF)
