# Supplementary material for: Calcium Dynamics of Ex Vivo Long-Term Cultured CD8+ T Cells Are Regulated by Changes in Redox Metabolism
Source: PLoS One. 2016 Aug 15;11(8):e0159248. doi: 10.1371/journal.pone.0159248 (PMC4985122; doi:10.1371/journal.pone.0159248)

S1 Fig. Example flow cytometry median calcium traces from a donor obtained at Day 4 and Day 24. After cold-binding with anti-CD3 and anti-CD28 as described in Materials and Methods, cells were sampled for 3 minutes by flow cytometry before addition of anti-mouse IgG. The average of the fluorescence ratio was calculated at 4 second intervals and then normalized to the average baseline value to provide a fold-change value.

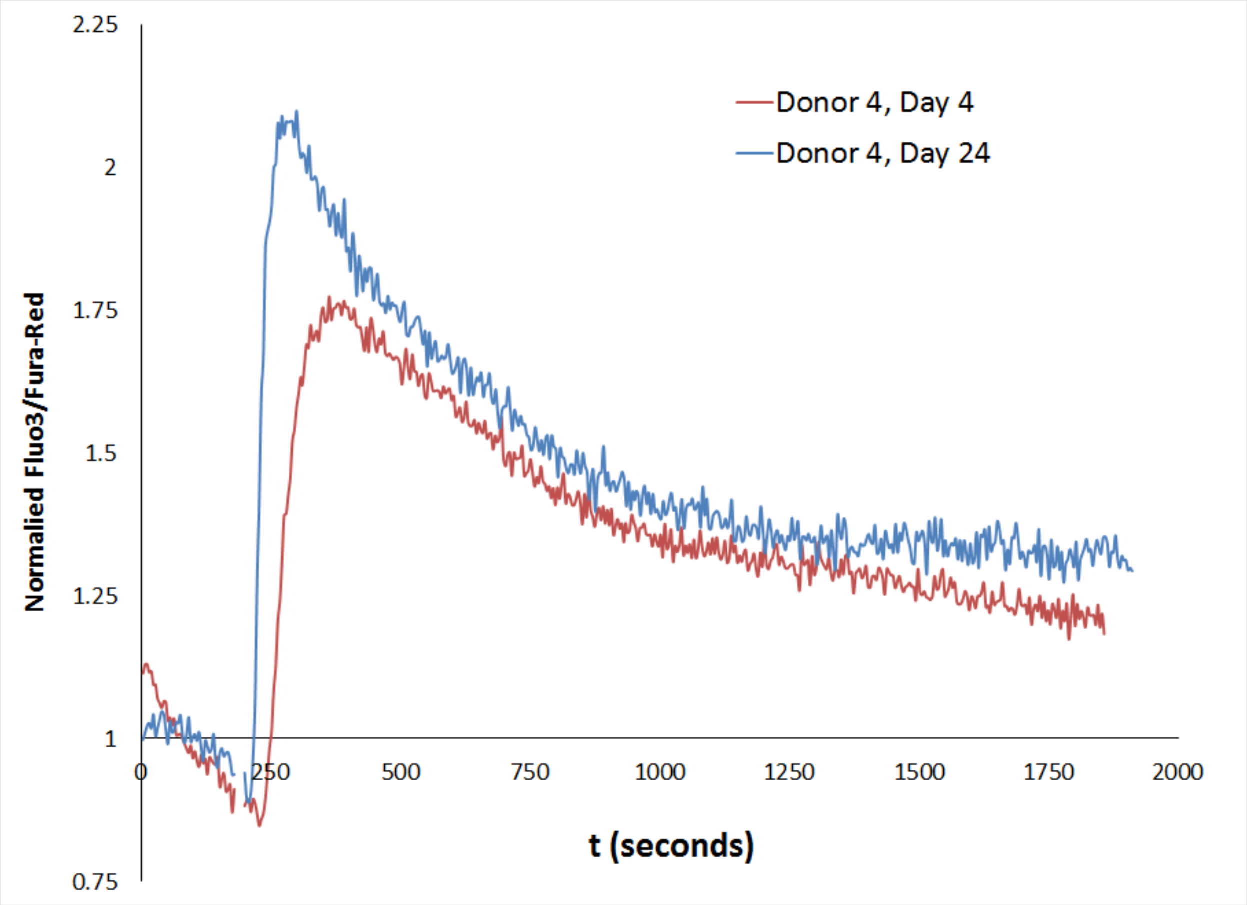

Supplement: S1 Fig — After cold-binding with anti-CD3 and anti-CD28 as described in Materials and Methods, cells were sampled for 3 minutes by flow cytometry before addition of anti-mouse IgG. The average of the fluorescence ratio was calculated at 4 second intervals and then normalized to the average baseline value to provide a fold-change value. (PDF) [file pone.0159248.s001.pdf]
