# Supplementary material for: Calcium Dynamics of Ex Vivo Long-Term Cultured CD8+ T Cells Are Regulated by Changes in Redox Metabolism
Source: PLoS One. 2016 Aug 15;11(8):e0159248. doi: 10.1371/journal.pone.0159248 (PMC4985122; doi:10.1371/journal.pone.0159248)

S2 Fig. Optimization of Jurkat T Cell Model using data obtained by no inhibitor, TMB-8, and EGTA conditions. Plots represent 17 different optimized parameter sets that were obtained from comparing the model prediction to experimental data.

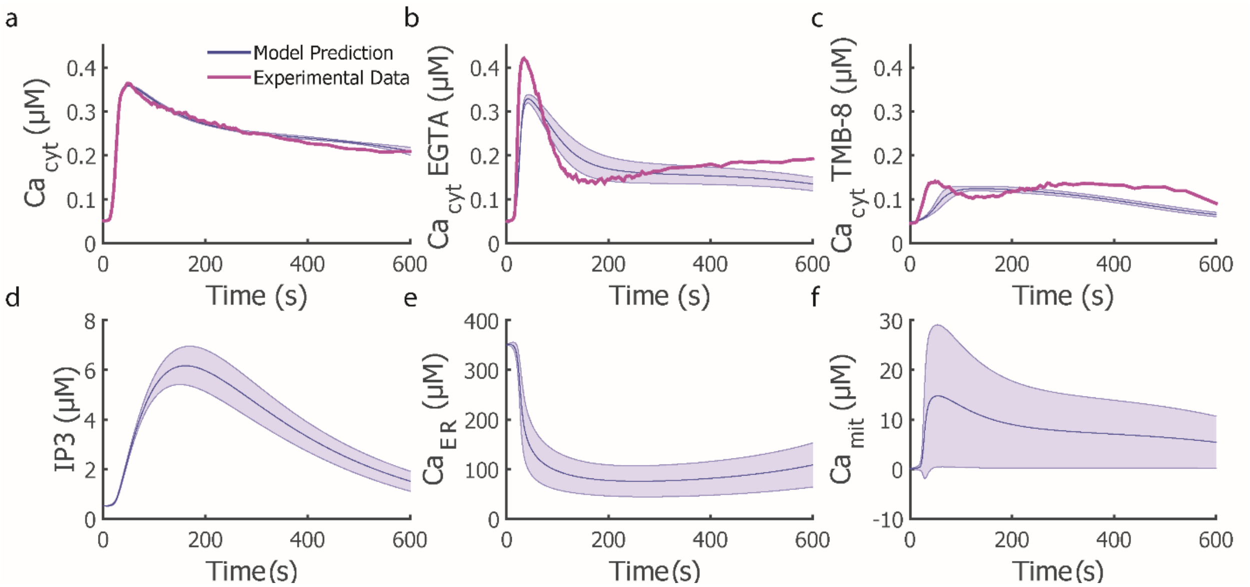

Supplement: S2 Fig — Plots represent 17 different optimized parameter sets that were obtained from comparing the model prediction to experimental data. (PDF) [file pone.0159248.s002.pdf]
