# Supplementary figures and images for: Calcium Dynamics of Ex Vivo Long-Term Cultured CD8+ T Cells Are Regulated by Changes in Redox Metabolism
Source: PLoS One. 2016 Aug 15;11(8):e0159248. doi: 10.1371/journal.pone.0159248 (PMC4985122; doi:10.1371/journal.pone.0159248)

**S3 Fig. Jurkat T Cell model behavior by parameter set used to fit Young CD8+ model**

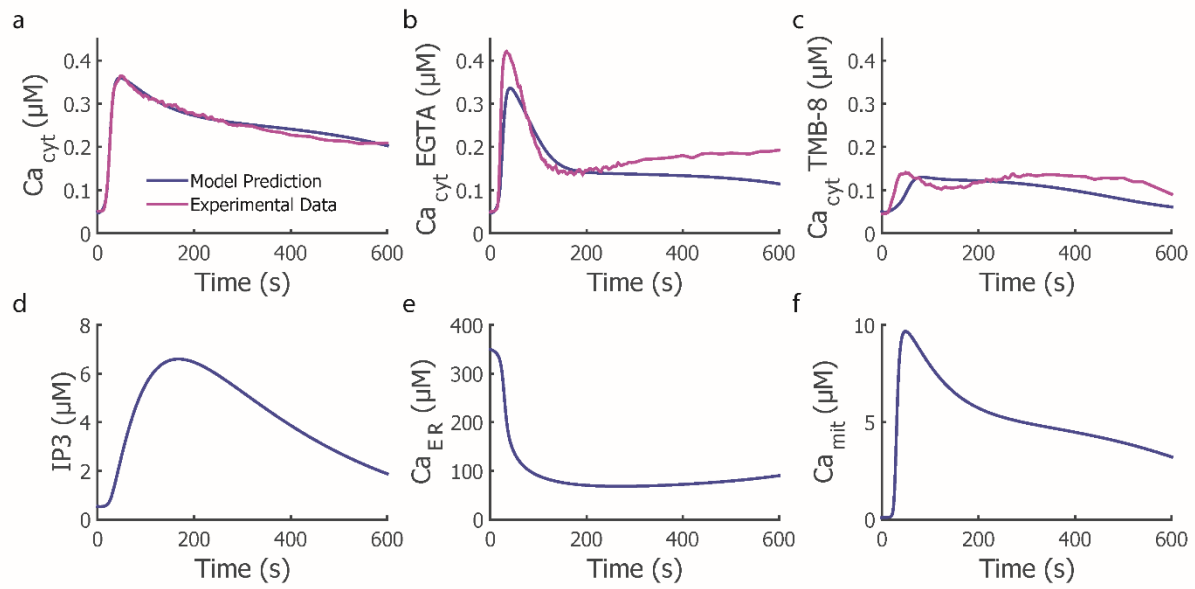

Supplement: S3 Fig — (PDF) [file pone.0159248.s003.pdf]

**S4 Fig. Optimized Young CD8<sup>+</sup> T Cell Model.**

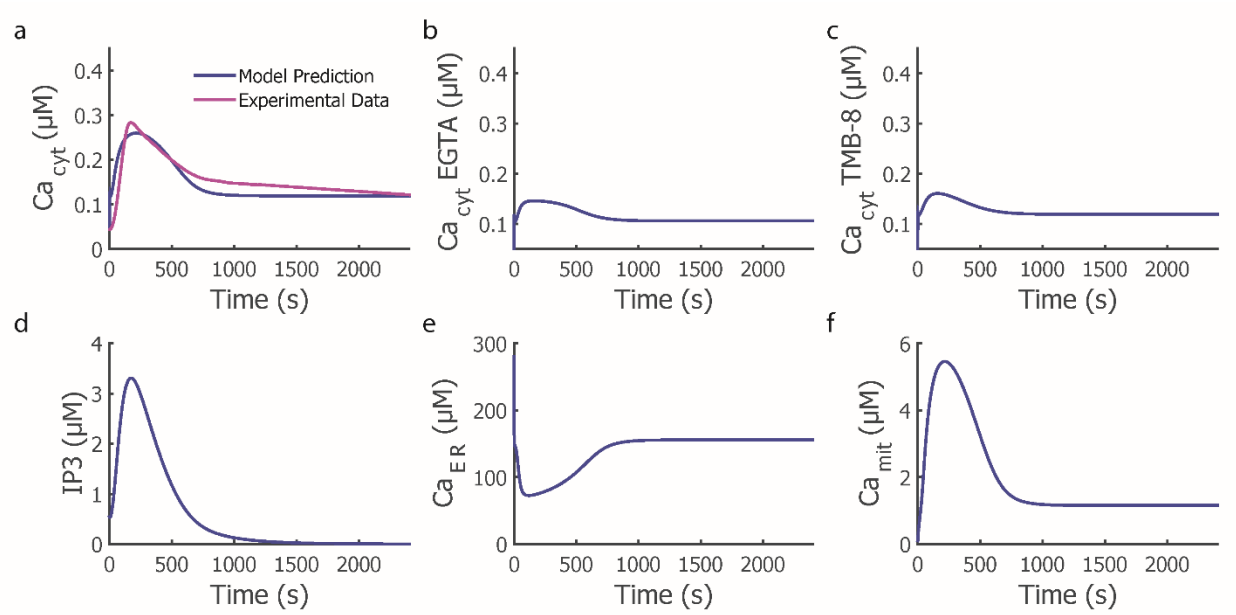

Supplement: S4 Fig — (PDF) [file pone.0159248.s004.pdf]

**S5 Fig. Best fit of Old CD8<sup>+</sup> T Cell Model varying only two parameters,  $V_{crac}$  and  $V_{pmca}$ .**

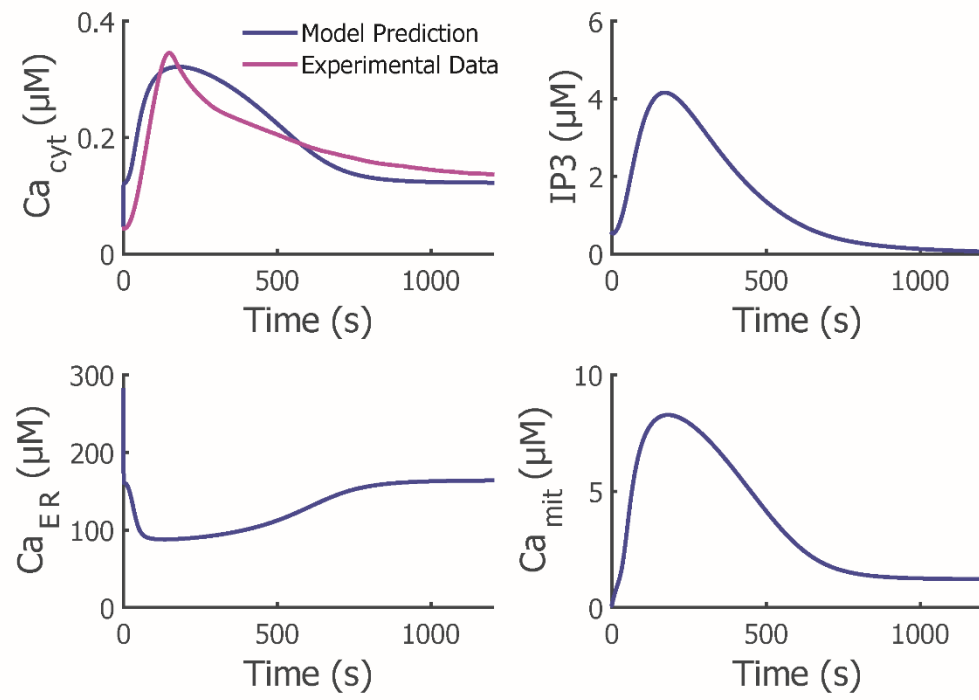

Supplement: S5 Fig — (PDF) [file pone.0159248.s005.pdf]

**S10 Fig. Expression of STIM1 in young and old primary human CD8+ T cells.**

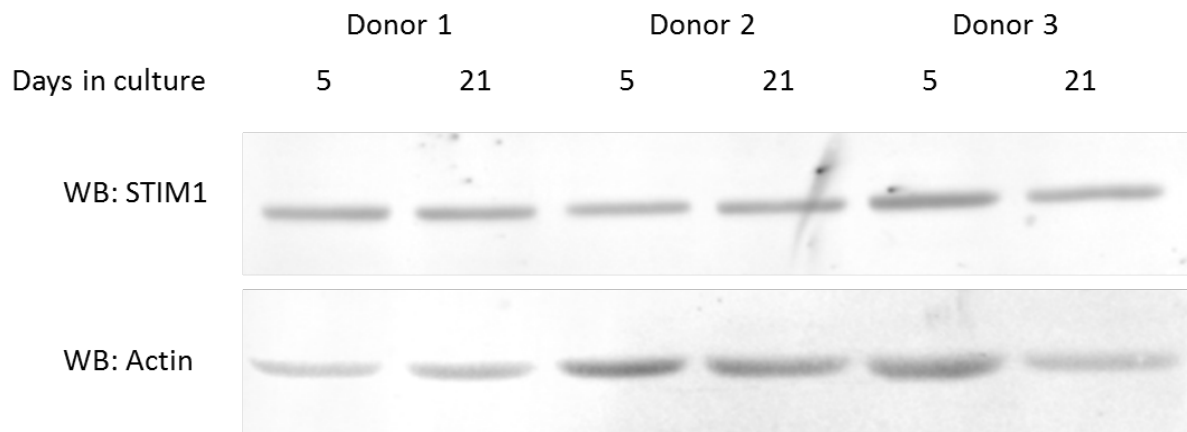

Supplement: S10 Fig — (PDF) [file pone.0159248.s010.pdf]
