# Supplementary material for: Calcium Dynamics of Ex Vivo Long-Term Cultured CD8+ T Cells Are Regulated by Changes in Redox Metabolism
Source: PLoS One. 2016 Aug 15;11(8):e0159248. doi: 10.1371/journal.pone.0159248 (PMC4985122; doi:10.1371/journal.pone.0159248)

S6 Fig. Best fit of Old CD8⁺ Young T Cell Model varying the seven parameters as identified in the sensitivity analysis of the Young CD8⁺ Young T Cell Model.

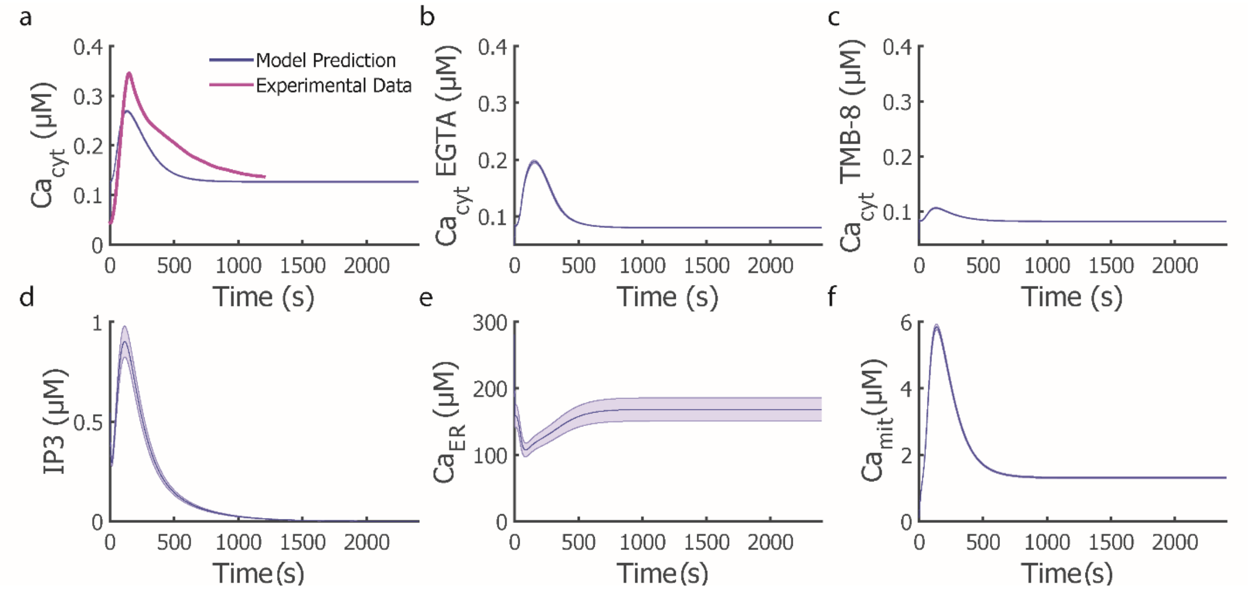

Supplement: S6 Fig — (PDF) [file pone.0159248.s006.pdf]
