# Supplementary material for: Calcium Dynamics of Ex Vivo Long-Term Cultured CD8+ T Cells Are Regulated by Changes in Redox Metabolism
Source: PLoS One. 2016 Aug 15;11(8):e0159248. doi: 10.1371/journal.pone.0159248 (PMC4985122; doi:10.1371/journal.pone.0159248)

S7 Fig. Varying K_{stim} from the Young CD8⁺ T Cell Model fit to investigate the effects on calcium traces. K_{stim} was varied +/- 20% the fit value of 178.

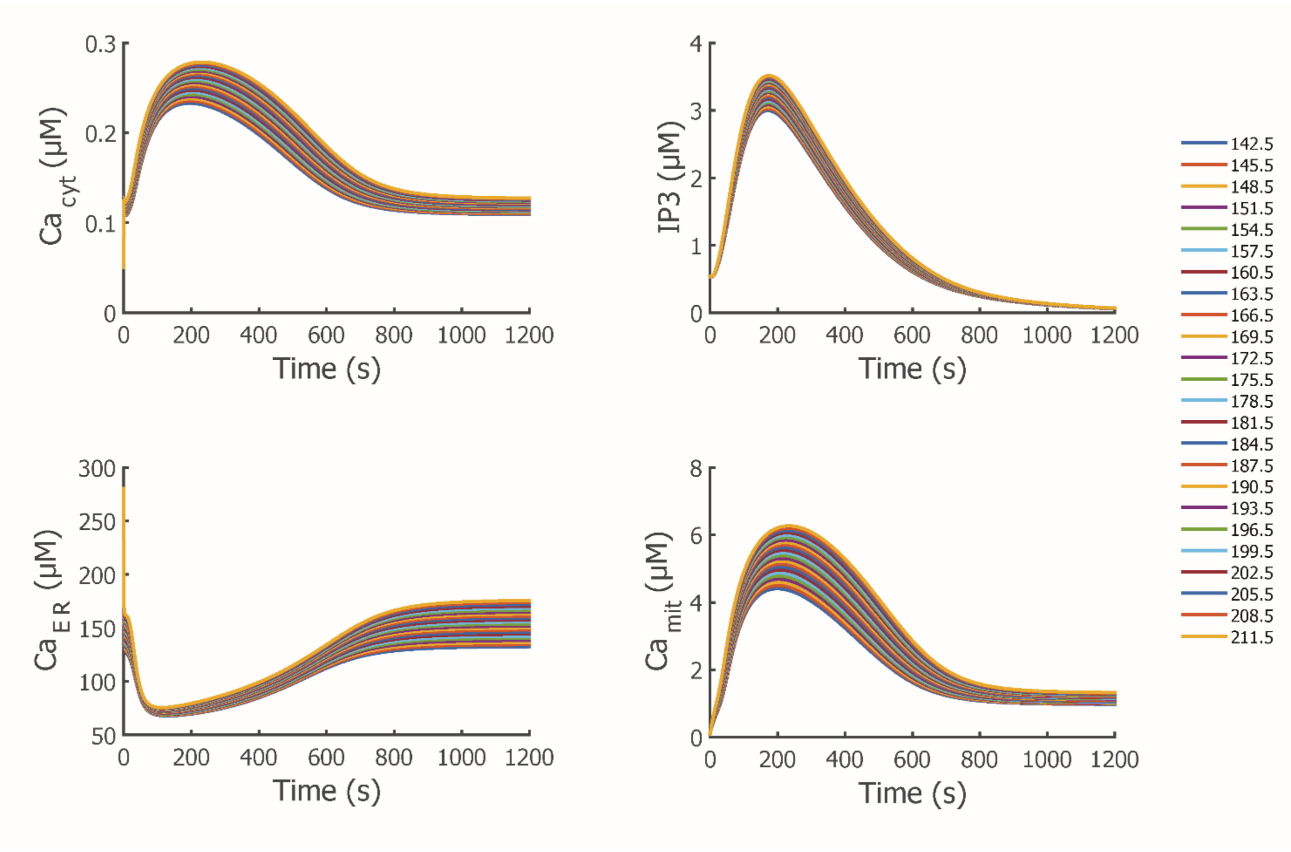

Supplement: S7 Fig — Kstim was varied +/- 20% the fit value of 178. (PDF) [file pone.0159248.s007.pdf]
