# Supplementary material for: Calcium Dynamics of Ex Vivo Long-Term Cultured CD8+ T Cells Are Regulated by Changes in Redox Metabolism
Source: PLoS One. 2016 Aug 15;11(8):e0159248. doi: 10.1371/journal.pone.0159248 (PMC4985122; doi:10.1371/journal.pone.0159248)

Supplemental Information

S1 Table. List of all oxidative stress and antioxidant PCR primer targets on the PCR array. Red genes represent targets that are not expressed in CD8+ T cells.

**ALB**	Albumin	**GLRX 2**	Glutaredoxin 2	**MTL5**	Metallothionein -like 5, testis-specific (tesmin)	**PTGS1**	Prostaglandin-endoperoxide synthase 1 (prostaglandin G/H synthase and cyclooxygenase)
**ALOX12**	Arachidonate 12-lipoxygenase	**GPR 156**	G protein-coupled receptor 156	**NCF1**	Neutrophil cytosolic factor 1	**PTGS2**	Prostaglandin-endoperoxide synthase 2 (prostaglandin G/H synthase and cyclooxygenase)
**ANGPTL 7**	Angiopoietin-like 7	**GPX1**	Glutathione peroxidase 1	**NCF2**	Neutrophil cytosolic factor 2	**PXDN**	Peroxidasin homolog (Drosophila)
**AOX1**	Aldehyde oxidase 1	**GPX2**	Glutathione peroxidase 2 (gastrointestinal)	**NME5**	Non-metastatic cells 5, protein expressed in (nucleoside-diphosphate kinase)	**PXDNL**	Peroxidasin homolog (Drosophila)-like
**APOE**	Apolipoprotein E	**GPX3**	Glutathione peroxidase 3 (plasma)	**NOS2**	Nitric oxide synthase 2, inducible	**RNF7**	Ring finger protein 7
**ATOX1**	ATX1 antioxidant protein 1 homolog (yeast)	**GPX4**	Glutathione peroxidase 4 (phospholipid hydroperoxidase)	**NOX5**	NADPH oxidase, EF-hand calcium binding domain 5	**SCARA 3**	Scavenger receptor class A, member 3
**BNIP3**	BCL2/adenoviru s E1B 19kDa interacting protein 3	**GPX5**	Glutathione peroxidase 5 (epididymal androgen-related protein)	**NUDT1**	Nudix (nucleoside diphosphate linked moiety X)-type motif 1	**SELS**	Selenoprotein S
**CAT**	Catalase	**GPX6**	Glutathione peroxidase 6 (olfactory)	**OXR1**	Oxidation resistance 1	**SEPP1**	Selenoprotein P, plasma, 1
**CCL5**	Chemokine (C-C motif) ligand 5	**GPX7**	Glutathione peroxidase 7	**OXSR1**	Oxidative-stress responsive 1	**SFTPD**	Surfactant protein D
**CCS**	Copper chaperone for superoxide dismutase	**GSR**	Glutathione reductase	**PDLIM 1**	PDZ and LIM domain 1	**SGK2**	Serum/glucocorticoid regulated kinase 2
**CSDE1**	Cold shock domain containing E1, RNA-binding	**GSS**	Glutathione synthetase	**IPCEF 1**	Interaction protein for cytohesin exchange factors 1	**SIRT2**	Sirtuin 2
**CYBA**	Cytochrome b-245, alpha	**GSTZ1**	Glutathione transferase zeta 1	**PNKP**	Polynucleotide kinase 3'-phosphatase	**SOD1**	Superoxide dismutase 1, soluble

	polypeptide						
**CYGB**	Cytoglobin	**GTF2I**	General transcription factor IIi	**PRDX1**	Peroxiredoxin 1	**SOD2**	Superoxide dismutase 2, mitochondrial
**DGKK**	Diacylglycerol kinase, kappa	**KRT1**	Keratin 1	**PRDX2**	Peroxiredoxin 2	**SOD3**	Superoxide dismutase 3, extracellular
**DHCR24**	24-dehydrocholesterol reductase	**LPO**	Lactoperoxidase	**PRDX3**	Peroxiredoxin 3	**SRXN1**	Sulfiredoxin 1
**DUOX1**	Dual oxidase 1	**MBL2**	Mannose-binding lectin (protein C) 2, soluble	**PRDX4**	Peroxiredoxin 4	**STK25**	Serine/threonine kinase 25
**DUOX2**	Dual oxidase 2	**MGST3**	Microsomal glutathione S-transferase 3	**PRDX5**	Peroxiredoxin 5	**TPO**	Thyroid peroxidase
**DUSP1**	Dual specificity phosphatase 1	**MPO**	Myeloperoxidase	**PRDX6**	Peroxiredoxin 6	**TTN**	Titin
**EPHX2**	Epoxide hydrolase 2, cytoplasmic	**MPV17**	MpV17 mitochondrial inner membrane protein	**PREX1**	Phosphatidylinositol-3,4,5-trisphosphate-dependent Rac exchange factor 1	**TXNDC2**	Thioredoxin domain containing 2 (spermatozoa)
**EPX**	Eosinophil peroxidase	**MSRA**	Methionine sulfoxide reductase A	**PRG3**	Proteoglycan 3	**TXNRD1**	Thioredoxin reductase 1
**FOXM1**	Forkhead box M1	**MT3**	Metallothionein 3	**PRNP**	Prion protein	**TXNRD2**	Thioredoxin reductase 2

S2 Table. Exhaustive list of fold changes and their corresponding p-values in targets expressed in CD8+ T cells. A fold change below 1 corresponds to a downregulation ($2^{-\Delta CT}$).

Symbol	Protein Name	Fold Change Old/Young	p-value
**PDLIM1**	PDZ and LIM domain 1	0.17	0.01
**ANGPTL7**	Angiopoietin-like 7	2.93	0.01
**MTL5**	Metallothionein-like 5, testis-specific (tesmin)	0.45	0.01
**NUDT1**	Nudix (nucleoside diphosphate linked moiety X)-type motif 1	0.99	0.01
**TTN**	Titin	2.14	0.01
**DUOX1**	Dual oxidase 1	1.68	0.03
**GPX3**	Glutathione peroxidase 3 (plasma)	2.55	0.04
**GLRX2**	Glutaredoxin 2	0.47	0.06
**PRNP**	Prion protein	1.45	0.07
**TXNRD1**	Thioredoxin reductase 1	0.31	0.08
**PRDX1**	Peroxiredoxin 1	0.74	0.13
**CCL5**	Chemokine (C-C motif) ligand 5	3.81	0.15
**TXNRD2**	Thioredoxin reductase 2	1.15	0.18
**GPX2**	Glutathione peroxidase 2 (gastrointestinal)	2.15	0.20
**SEPP1**	Selenoprotein P, plasma, 1	0.34	0.21
**ATOX1**	ATX1 antioxidant protein 1 homolog (yeast)	0.50	0.22
**BNIP3**	BCL2/adenovirus E1B 19kDa interacting protein 3	1.12	0.23
**CCS**	Copper chaperone for superoxide dismutase	0.22	0.23
**PX1**	Glutathione peroxidase 1	0.18	0.23
**PREX1**	Phosphatidylinositol-3,4,5-trisphosphate-dependent Rac exchange factor 1	0.90	0.24
**PRDX5**	Peroxiredoxin 5	0.80	0.29
**CSDE1**	Cold shock domain containing E1, RNA-binding	0.49	0.30
**SIRT2**	Sirtuin 2	1.25	0.32
**FOXM1**	Forkhead box M1	1.78	0.33
**GTF2I**	General transcription factor IIi	0.52	0.34
**GSS**	Glutathione synthetase	0.26	0.37
**NCF1**	Neutrophil cytosolic factor 1	9.32	0.42
**ALOX12**	Arachidonate 12-lipoxygenase	0.60	0.46
**STK25**	Serine/threonine kinase 25	4.65	0.46
**GPX4**	Glutathione peroxidase 4 (phospholipid hydroperoxidase)	0.02	0.47
**SELS**	Selenoprotein S	0.10	0.48
**MPV17**	MpV17 mitochondrial inner membrane protein	0.57	0.49
**MGST3**	Microsomal glutathione S-transferase 3	0.49	0.50
**GSR**	Glutathione reductase	0.73	0.51
**PRDX2**	Peroxiredoxin 2	0.75	0.51
**SOD1**	Superoxide dismutase 1, soluble	6.52	0.51
**SRXN1**	Sulfiredoxin 1	0.12	0.52
**CAT**	Catalase	122.49	0.56
**MSRA**	Methionine sulfoxide reductase A	0.79	0.57
**MT3**	Metallothionein 3	0.28	0.59
**NCF2**	Neutrophil cytosolic factor 2	1.49	0.59

**OXR1**	Oxidation resistance 1	0.02	0.59
**GPX7**	Glutathione peroxidase 7	0.17	0.60
**EPX**	Eosinophil peroxidase	0.83	0.64
**CYBA**	P22-phox	0.41	0.65
**GSTZ1**	Glutathione transferase zeta 1	1.89	0.65
**PRDX6**	Peroxiredoxin 6	3.93	0.65
**IPCEF1**	Interaction protein for cytohesin exchange factors 1	0.65	0.66
**PRDX4**	Peroxiredoxin 4	2.87	0.70
**SOD2**	Superoxide dismutase 2, mitochondrial	0.33	0.70
**DUSP1**	Dual specificity phosphatase 1	4.56	0.71
**RNF7**	Ring finger protein 7	6.55	0.71
**SFTPD**	Surfactant protein D	3.35	0.72
**DHCR24**	24-dehydrocholesterol reductase	0.57	0.82
**PRDX3**	Peroxiredoxin 3	1.60	0.84
**OXSR1**	Oxidation resistance 1	2.20	0.95
**PNKP**	Polynucleotide kinase 3'-phosphatase	1.59	0.98

S3 Table S3. Normalized mRNA levels of individual genes expressed in young CD8+ T cells, ranked in descending order of expression (n=6).

Symbol	Protein Name	Mean ΔCt ± SEM	% CV
**CYBA**	P22-phox	3.77 ± 0.4	10.64
**CCL5**	Chemokine (C-C motif) ligand 5	3.58 ± 0.68	19.06
**SOD1**	Superoxide dismutase 1, soluble	4.19 ± 0.23	5.56
**GPX4**	Glutathione peroxidase 4 (phospholipid hydroperoxidase)	4.38 ± 0.40	9.24
**PRDX6**	Peroxiredoxin 6	4.60 ± 0.30	6.48
**GTF2I**	General transcription factor IIi	4.79 ± 0.28	5.93
**PRDX3**	Peroxiredoxin 3	4.61 ± 0.46	9.98
**PRDX2**	Peroxiredoxin 2	4.78 ± 0.35	7.24
**GPX1**	Glutathione peroxidase 1	5.17 ± 0.23	4.48
**CSDE1**	Cold shock domain containing E1, RNA-binding	5.19 ± 0.23	4.48
**FOXM1**	Forkhead box M1	5.39 ± 0.18	3.44
**PRDX5**	Peroxiredoxin 5	5.38 ± 0.28	5.25
**ATOX1**	ATX1 antioxidant protein 1 homolog (yeast)	5.60 ± 0.21	3.70
**TXNRD1**	Thioredoxin reductase 1	6.17 ± 0.08	1.33
**DHCR24**	24-dehydrocholesterol reductase	6.38 ± 0.18	2.83
**RNF7**	Ring finger protein 7	6.58 ± 0.21	3.13
**SOD2**	Superoxide dismutase 2, mitochondrial	6.59 ± 0.21	3.12
**OXSR1**	Oxidative-stress responsive 1	6.98 ± 0.10	1.47
**CAT**	Catalase	6.78 ± 0.4	5.92
**PRDX4**	Peroxiredoxin 4	6.99 ± 0.36	5.18
**MGST3**	Microsomal glutathione S-transferase 3	7.18 ± 0.23	3.22
**MPV17**	MpV17 mitochondrial inner membrane protein	7.18 ± 0.23	3.21
**GLRX2**	Glutaredoxin 2	7.39 ± 0.18	2.49
**GSS**	Glutathione synthetase	7.37 ± 0.28	3.81
**BNIP3**	BCL2/adenovirus E1B 19kDa interacting protein 3	7.38 ± 0.34	4.62
**SELS**	Selenoprotein S	7.78 ± 0.28	3.62
**STK25**	Serine/threonine kinase 25	7.98 ± 0.10	1.24
**TXNRD2**	Thioredoxin reductase 2	7.79 ± 0.34	4.43
**NUDT1**	Nudix (nucleoside diphosphate linked moiety X)-type motif 1	8.18 ± 0.09	1.04
**PNKP**	Polynucleotide kinase 3'-phosphatase	7.99 ± 0.31	3.89
**GPX7**	Glutathione peroxidase 7	8.00 ± 0.31	3.89
**OXR1**	Oxidation resistance 1	8.19 ± 0.23	2.85
**PRDX1**	Peroxiredoxin 1	8.19 ± 0.30	3.70
**CCS**	Copper chaperone for superoxide dismutase	8.80 ± 0.19	2.13
**IPCEF1**	Interaction protein for cytohesin exchange factors 1	8.57 ± 0.58	6.78
**MTL5**	Metallothionein-like 5, testis-specific (tesmin)	8.97 ± 0.31	3.46
**SIRT2**	Sirtuin 2	9.01 ± 0.38	4.20
**PREX1**	Phosphatidylinositol-3,4,5-trisphosphate-dependent Rac exchange factor 1	9.18 ± 0.23	2.49
**GSTZ1**	Glutathione transferase zeta 1	9.19 ± 0.37	4.07
**NCF2**	Neutrophil cytosolic factor 2	9.38 ± 0.39	4.20
**NCF1**	Neutrophil cytosolic factor 1	9.00 ± 0.89	9.91

**SRXN1**	Sulfiredoxin 1	10.18 ± 0.23	2.26
**MSRA**	Methionine sulfoxide reductase A	10.18 ± 0.3	2.93
**DUSP1**	Dual specificity phosphatase 1	10.19 ± 0.43	4.19
**GPX3**	Glutathione peroxidase 3 (plasma)	10.37 ± 0.49	4.76
**SEPP1**	Selenoprotein P, plasma, 1	10.57 ± 0.29	2.80
**PRNP**	Prion protein	11.01 ± 0.10	0.90
**GSR**	Glutathione reductase	10.78 ± 0.34	3.17
**PDLIM1**	PDZ and LIM domain 1	10.98 ± 0.3	2.79
**TTN**	Titin	11.19 ± 0.42	3.79
**MT3**	Metallothionein 3	11.4 ± 0.73	6.40
**ANGPTL7**	Angiopoietin-like 7	12.17 ± 0.23	1.87
**ALOX12**	Arachidonate 12-lipoxygenase	12.17 ± 0.42	3.48
**EPX**	Eosinophil peroxidase	12.58 ± 0.41	3.29
**DUOX1**	Dual oxidase 1	12.78 ± 0.34	2.68
**SFTPD**	Surfactant protein D	12.57 ± 0.76	6.07
**PXDN**	Peroxidasin homolog (Drosophila)	13.21 ± 0.62	4.70
**GPX2**	Glutathione peroxidase 2 (gastrointestinal)	13.99 ± 0.47	3.36

S1 Fig. Example flow cytometry median calcium traces from a donor obtained at Day 4 and Day 24. After cold-binding with anti-CD3 and anti-CD28 as described in Materials and Methods, cells were sampled for 3 minutes by flow cytometry before addition of anti-mouse IgG. The average of the fluorescence ratio was calculated at 4 second intervals and then normalized to the average baseline value to provide a fold-change value.

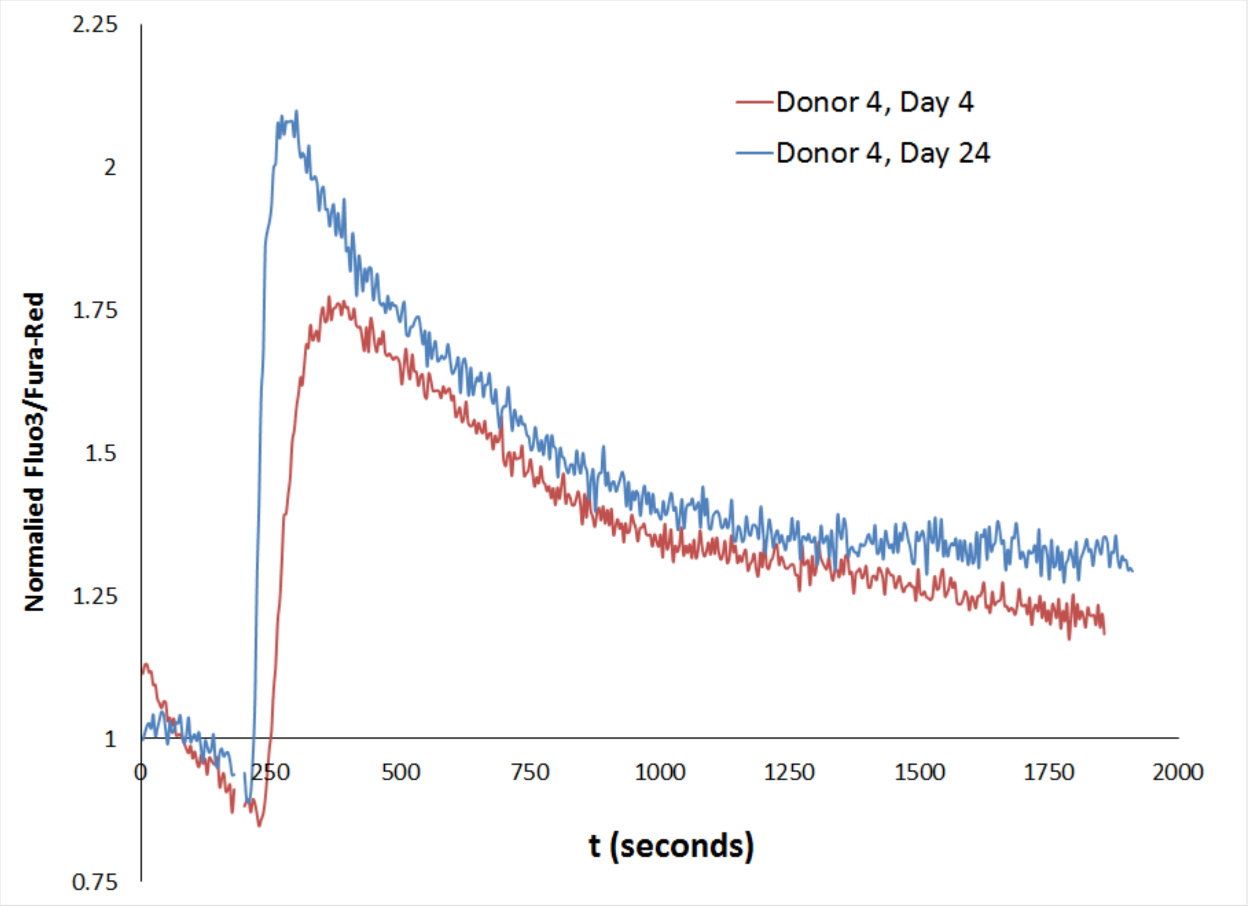

S2 Fig. Optimization of Jurkat T Cell Model using data obtained by no inhibitor, TMB-8, and EGTA conditions. Plots represent 17 different optimized parameter sets that were obtained from comparing the model prediction to experimental data.

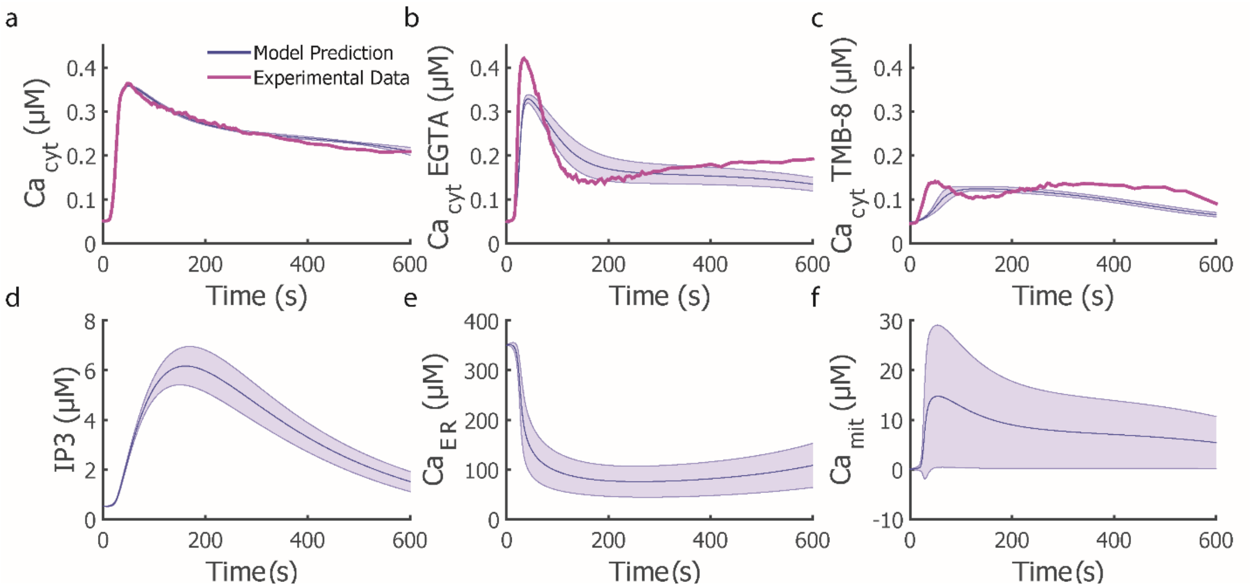

S3 Fig. Jurkat T Cell model behavior by parameter set used to fit Young CD8+ model

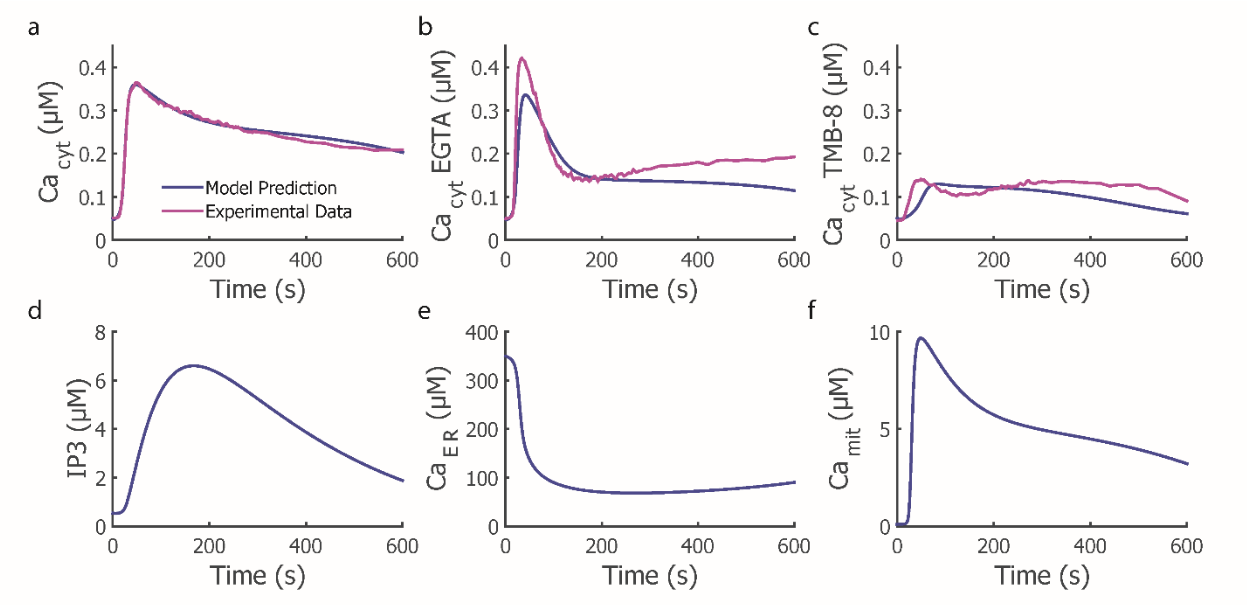

S4 Table. Optimized parameter set obtained from the Jurkat T Cell Model fitting used for the seeding the initial population of parameter values for the genetic algorithm optimization of the Young CD8^{+} T Cell Model to experimental data.

Parameter	Jurkat T cells
$\boldsymbol{\beta_i}$*	0.056
$\boldsymbol{\beta_{er}}$*	0.049
$\boldsymbol{\beta_{mit}}$	0.033
$\boldsymbol{\rho_{er}}$	0.015
$\boldsymbol{\rho_{mit}}$	0.08
$\boldsymbol{k_{PLCact}}$	0.0033 s^{-1}
$\boldsymbol{k_{PLCdeact}}$	0.042 s^{-1}
$\boldsymbol{k_{IP3prod}}$	0.48 $\mu M^{-1}\ s^{-1}$
$\boldsymbol{k_{IP3deg}}$*	0.010 s^{-1}
$\boldsymbol{V_{IP3}}$	4.0 s^{-1}
$\boldsymbol{K_{IP3}}$	0.57 μM
$\boldsymbol{K_{act}}$	0.13 μM
$\boldsymbol{A}$	0.079
$\boldsymbol{K_{inh}}$	1 μM
$\boldsymbol{K_{IP3inh}}$*	0.82 μM
$\boldsymbol{K_{ERleak}}$*	0.0043 s^{-1}
$\boldsymbol{V_{serca}}$*	112.75 $\mu M\ s^{-1}$
$\boldsymbol{K_{serca}}$	0.43 μM
$\boldsymbol{V_{mitin}}$	388.6 $\mu M\ s^{-1}$
$\boldsymbol{K_{mitin}}$	0.81 μM
$\boldsymbol{V_{mitout}}$*	188.9 $\mu M\ s^{-1}$
$\boldsymbol{K_{mitout}}$*	4.03 μM
$\boldsymbol{V_{crac}}$	2.4 $\mu M\ s^{-1}$
$\boldsymbol{K_{soc}}$*	363.5 μM
$\boldsymbol{K_{stim}}$	178.1 μM

$\boldsymbol{K_{PMleak}}$	1.1e-6 s^{-1}
$\boldsymbol{V_{pmca}}$*	2.14 μM s^{-1}
$\boldsymbol{K_{pmca}}$	0.11 μM

S5 Table. Optimized parameter set obtained from fitting the Young CD8$^+$ T Cell Model to experimental data. This parameter set was used for all sensitivity analysis performed on the Young CD8$^+$ T Cell Model.

**Parameter**	**Primary CD8$^+$ T cells**
$\boldsymbol{\beta_i}$*	0.047
$\boldsymbol{\beta_{er}}$*	0.98
$\boldsymbol{\beta_{mit}}$	0.033
$\boldsymbol{\rho_{er}}$	0.015
$\boldsymbol{\rho_{mit}}$	0.08
$\boldsymbol{k_{PLCact}}$	0.0033 s^{-1}
$\boldsymbol{k_{PLCdeact}}$	0.042 s^{-1}
$\boldsymbol{k_{IP3prod}}$	0.48 μM^{-1} s^{-1}
$\boldsymbol{k_{IP3deg}}$*	0.018 μs^{-1}
$\boldsymbol{V_{IP3}}$	4.0 μs^{-1}
$\boldsymbol{K_{IP3}}$	0.57 μM
$\boldsymbol{K_{act}}$	0.13 μM
$\boldsymbol{A}$	0.079
$\boldsymbol{K_{inh}}$	1 μM
$\boldsymbol{K_{IP3inh}}$*	1.5 μM
$\boldsymbol{K_{ERleak}}$*	0.048 s^{-1}
$\boldsymbol{V_{serca}}$*	103.88 μM s^{-1}
$\boldsymbol{K_{serca}}$	0.43 μM
$\boldsymbol{V_{mitin}}$	388.6 μM s^{-1}
$\boldsymbol{K_{mitin}}$	0.81 μM
$\boldsymbol{V_{mitout}}$*	244.7 μM s^{-1}
$\boldsymbol{K_{mitout}}$*	4.7 μM
$\boldsymbol{V_{crac}}$	2.4 μM s^{-1}

K_{soc}*	358.8 μM
K_{stim}	178.1 μM
K_{PMleak}	1.1e-6 s^{-1}
V_{pmca}*	2.08 μM s^{-1}
K_{pmca}	0.11 μM

S4 Fig. Optimized Young CD8+ T Cell Model.

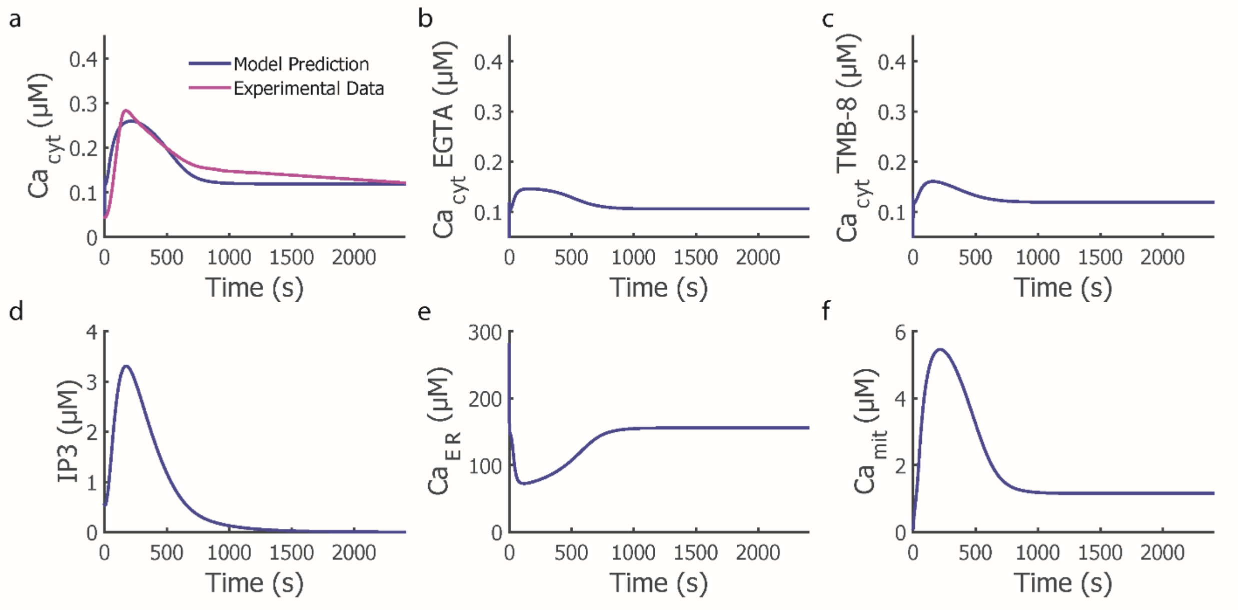

S5 Fig. Best fit of Old CD8+ T Cell Model varying only two parameters, V_{crac} and V_{pmca}.

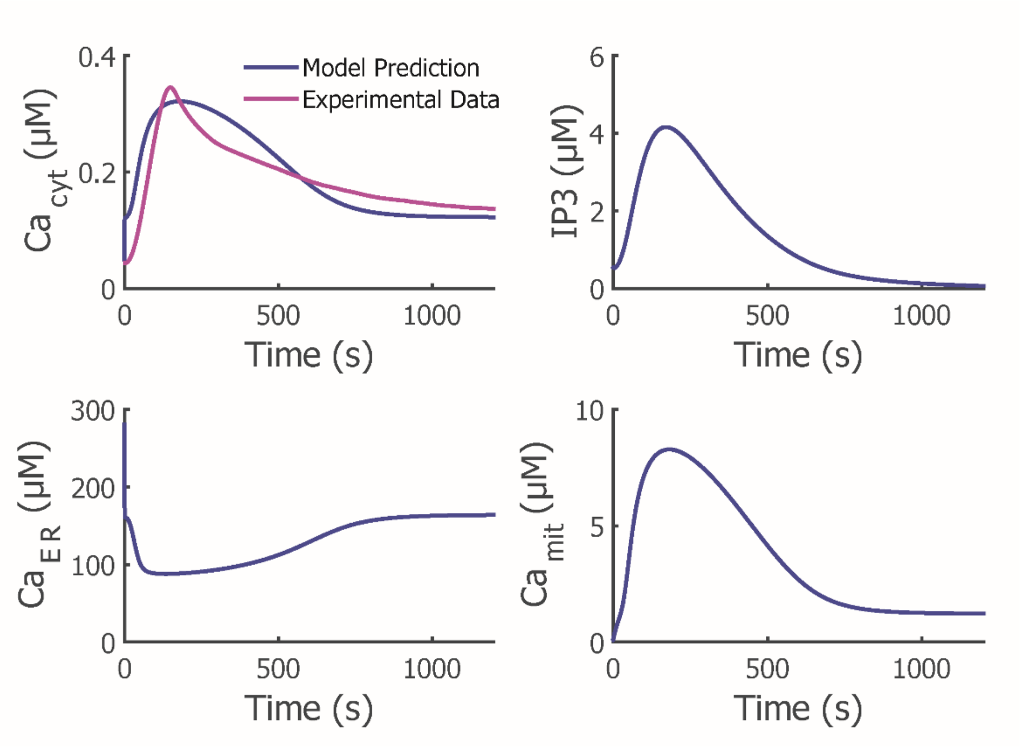

S6 Fig. Best fit of Old CD8+ Young T Cell Model varying the seven parameters as identified in the sensitivity analysis of the Young CD8+ Young T Cell Model.

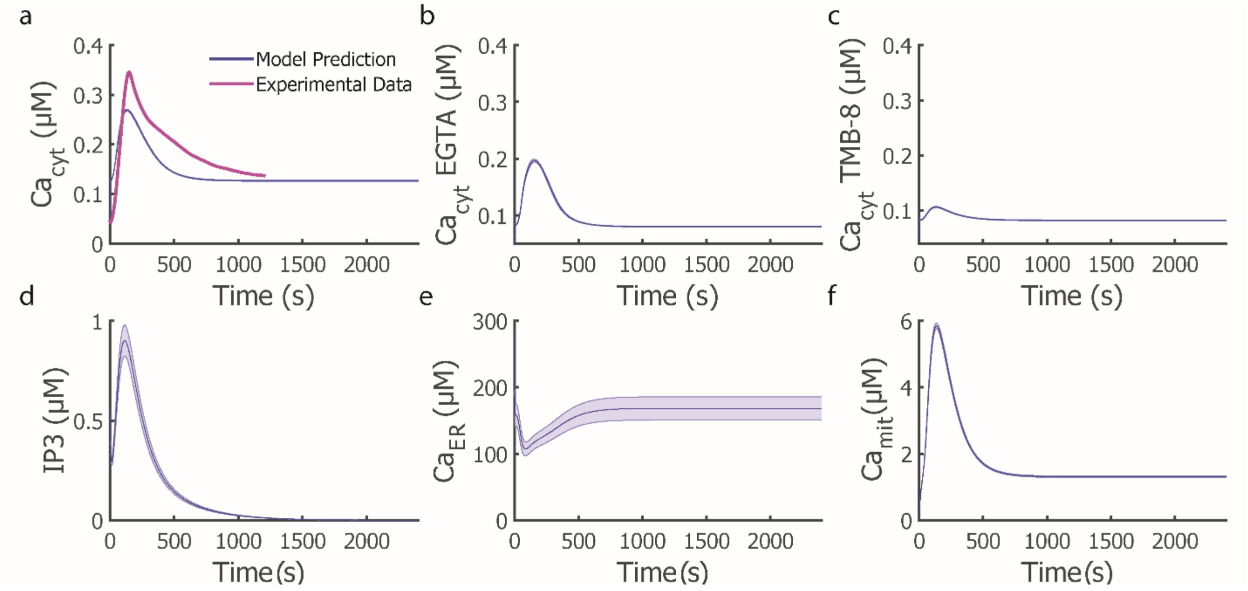

S7 Fig. Varying K_{stim} from the Young CD8^{+} T Cell Model fit to investigate the effects on calcium traces. K_{stim} was varied +/- 20% the fit value of 178.

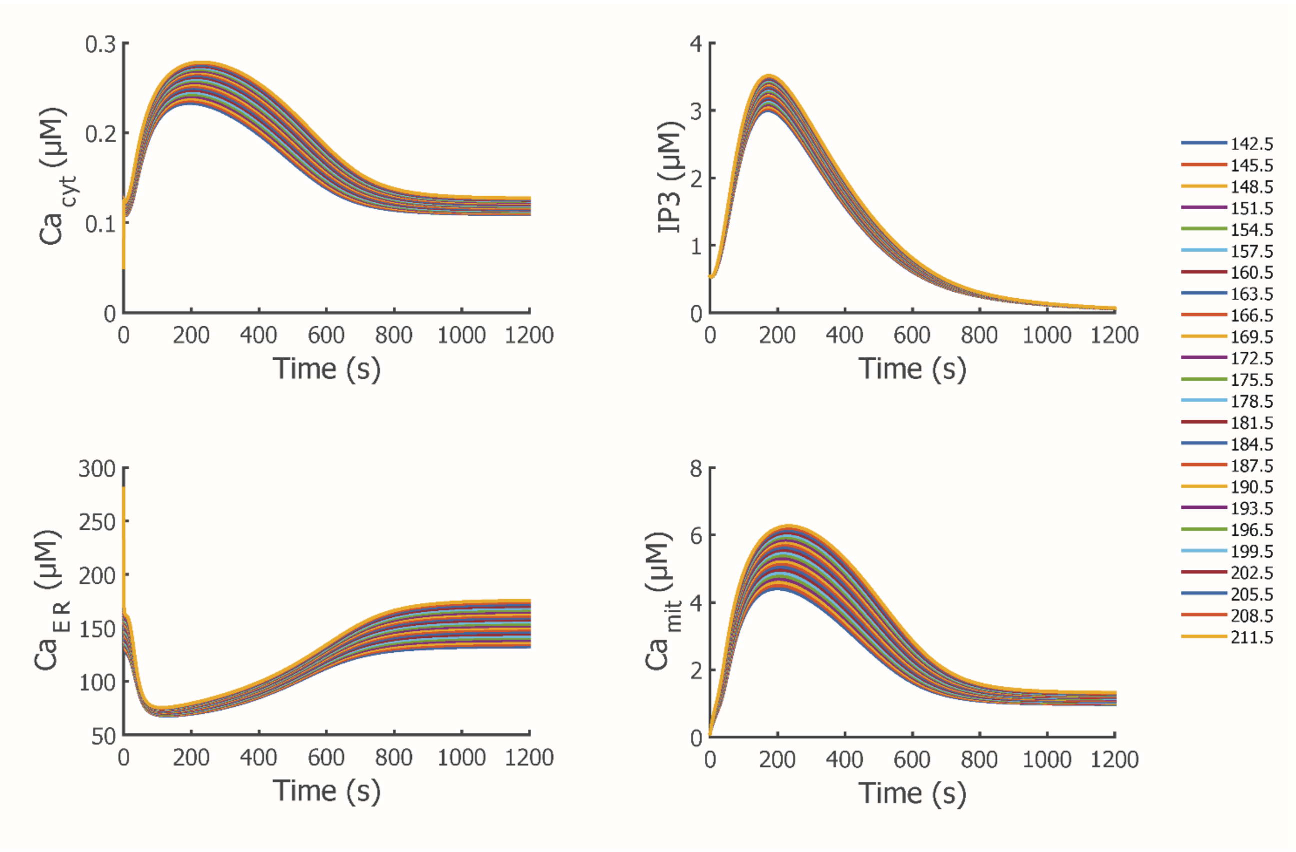

S8 Fig. Varying V_{crac} from the Young CD8⁺ T Cell Model to investigate the effects on calcium traces. V_{crac} was varied +/- 20% the fit value of 2.37.

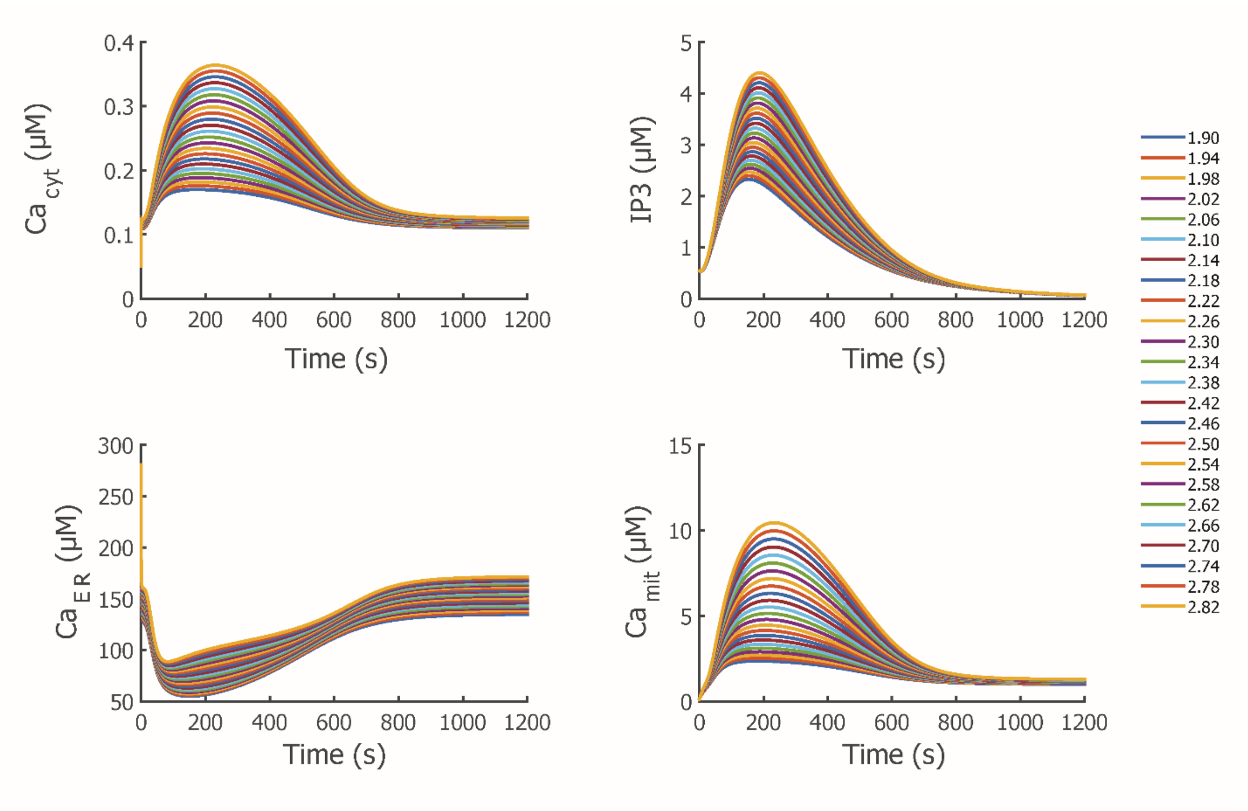

S9 Fig. Validation of RT-PCR results with Duox 1 expression. a) Representative Western Blot. b) Quantification of the Western Blots. Protein levels are normalized to the young cells protein expression level. * $p<0.05$ (paired 2-tail t-test).

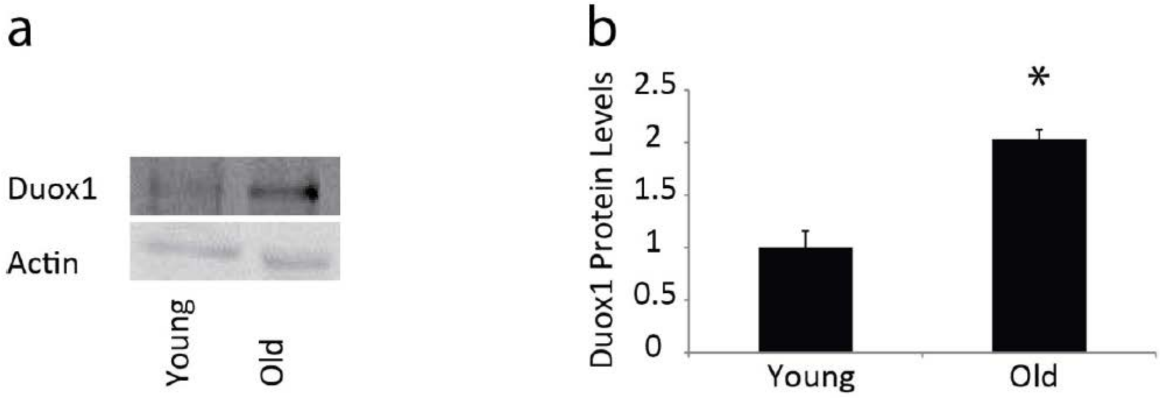

S10 Fig. Expression of STIM1 in young and old primary human CD8+ T cells.

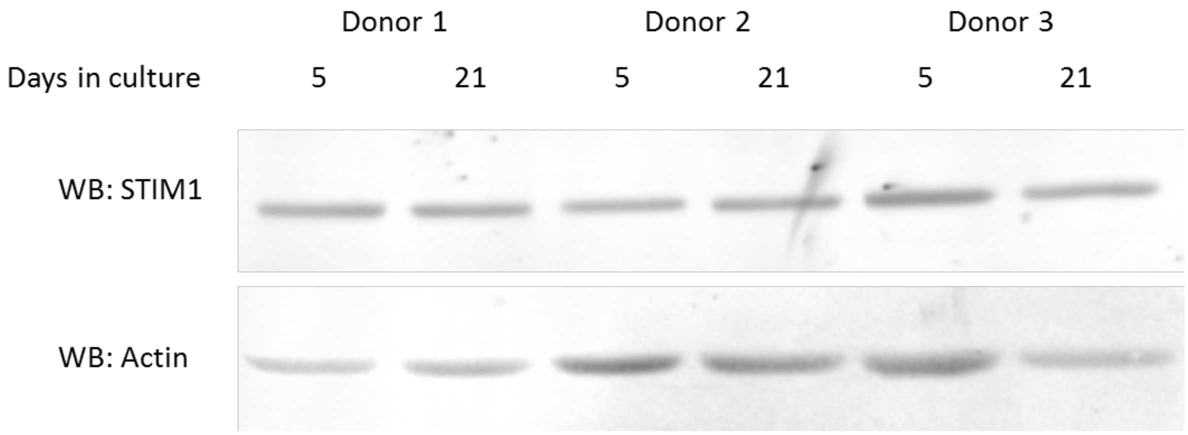

Supplement: S8 Fig — Vcrac was varied +/- 20% the fit value of 2.37. (PDF) [file pone.0159248.s008.pdf]
