# Supplementary material for: Calcium Dynamics of Ex Vivo Long-Term Cultured CD8+ T Cells Are Regulated by Changes in Redox Metabolism
Source: PLoS One. 2016 Aug 15;11(8):e0159248. doi: 10.1371/journal.pone.0159248 (PMC4985122; doi:10.1371/journal.pone.0159248)

S9 Fig. Validation of RT-PCR results with Duox 1 expression. a) Representative Western Blot. b) Quantification of the Western Blots. Protein levels are normalized to the young cells protein expression level. * p<0.05 (paired 2-tail t-test).

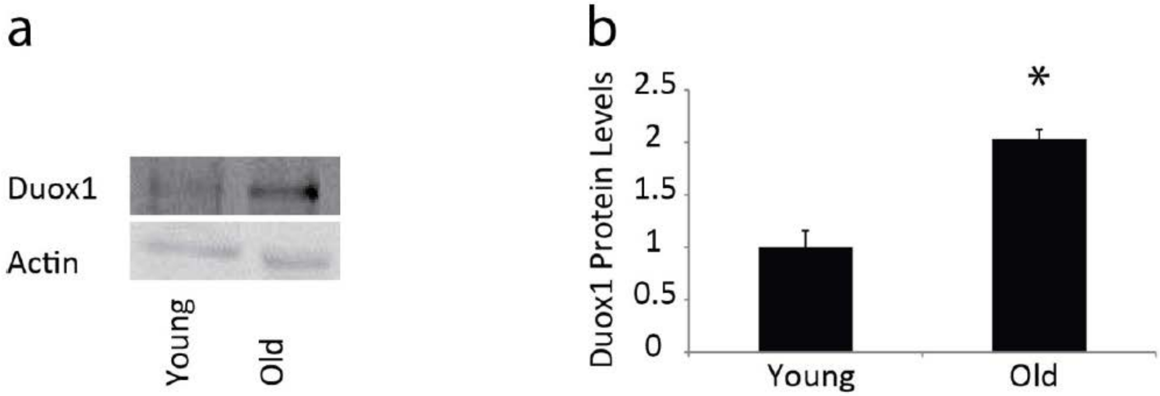

Supplement: S9 Fig — a) Representative Western Blot. b) Quantification of the Western Blots. Protein levels are normalized to the young cells protein expression level. * p<0.05 (paired 2-tail t-test). (PDF) [file pone.0159248.s009.pdf]
