# Supplementary material for: Calcium Dynamics of Ex Vivo Long-Term Cultured CD8+ T Cells Are Regulated by Changes in Redox Metabolism
Source: PLoS One. 2016 Aug 15;11(8):e0159248. doi: 10.1371/journal.pone.0159248 (PMC4985122; doi:10.1371/journal.pone.0159248)
Supplement: S1 Table — Red genes represent targets that are not expressed in CD8+ T cells. (PDF) [file pone.0159248.s011.pdf]

Supplemental Information

S1 Table. List of all oxidative stress and antioxidant PCR primer targets on the PCR array. Red genes represent targets that are not expressed in CD8+ T cells.

**ALB**	Albumin	**GLRX 2**	Glutaredoxin 2	**MTL5**	Metallothionein -like 5, testis-specific (tesmin)	**PTGS1**	Prostaglandin-endoperoxide synthase 1 (prostaglandin G/H synthase and cyclooxygenase)
**ALOX12**	Arachidonate 12-lipoxygenase	**GPR 156**	G protein-coupled receptor 156	**NCF1**	Neutrophil cytosolic factor 1	**PTGS2**	Prostaglandin-endoperoxide synthase 2 (prostaglandin G/H synthase and cyclooxygenase)
**ANGPTL 7**	Angiopoietin-like 7	**GPX1**	Glutathione peroxidase 1	**NCF2**	Neutrophil cytosolic factor 2	**PXDN**	Peroxidasin homolog (Drosophila)
**AOX1**	Aldehyde oxidase 1	**GPX2**	Glutathione peroxidase 2 (gastrointestinal)	**NME5**	Non-metastatic cells 5, protein expressed in (nucleoside-diphosphate kinase)	**PXDNL**	Peroxidasin homolog (Drosophila)-like
**APOE**	Apolipoprotein E	**GPX3**	Glutathione peroxidase 3 (plasma)	**NOS2**	Nitric oxide synthase 2, inducible	**RNF7**	Ring finger protein 7
**ATOX1**	ATX1 antioxidant protein 1 homolog (yeast)	**GPX4**	Glutathione peroxidase 4 (phospholipid hydroperoxidase)	**NOX5**	NADPH oxidase, EF-hand calcium binding domain 5	**SCARA 3**	Scavenger receptor class A, member 3
**BNIP3**	BCL2/adenoviru s E1B 19kDa interacting protein 3	**GPX5**	Glutathione peroxidase 5 (epididymal androgen-related protein)	**NUDT1**	Nudix (nucleoside diphosphate linked moiety X)-type motif 1	**SELS**	Selenoprotein S
**CAT**	Catalase	**GPX6**	Glutathione peroxidase 6 (olfactory)	**OXR1**	Oxidation resistance 1	**SEPP1**	Selenoprotein P, plasma, 1
**CCL5**	Chemokine (C-C motif) ligand 5	**GPX7**	Glutathione peroxidase 7	**OXSR1**	Oxidative-stress responsive 1	**SFTPD**	Surfactant protein D
**CCS**	Copper chaperone for superoxide dismutase	**GSR**	Glutathione reductase	**PDLIM 1**	PDZ and LIM domain 1	**SGK2**	Serum/glucocorticoid regulated kinase 2
**CSDE1**	Cold shock domain containing E1, RNA-binding	**GSS**	Glutathione synthetase	**IPCEF 1**	Interaction protein for cytohesin exchange factors 1	**SIRT2**	Sirtuin 2
**CYBA**	Cytochrome b-245, alpha	**GSTZ1**	Glutathione transferase zeta 1	**PNKP**	Polynucleotide kinase 3'-phosphatase	**SOD1**	Superoxide dismutase 1, soluble

	polypeptide						
**CYGB**	Cytoglobin	**GTF2I**	General transcription factor IIi	**PRDX1**	Peroxiredoxin 1	**SOD2**	Superoxide dismutase 2, mitochondrial
**DGKK**	Diacylglycerol kinase, kappa	**KRT1**	Keratin 1	**PRDX2**	Peroxiredoxin 2	**SOD3**	Superoxide dismutase 3, extracellular
**DHCR24**	24-dehydrocholesterol reductase	**LPO**	Lactoperoxidase	**PRDX3**	Peroxiredoxin 3	**SRXN1**	Sulfiredoxin 1
**DUOX1**	Dual oxidase 1	**MBL2**	Mannose-binding lectin (protein C) 2, soluble	**PRDX4**	Peroxiredoxin 4	**STK25**	Serine/threonine kinase 25
**DUOX2**	Dual oxidase 2	**MGST3**	Microsomal glutathione S-transferase 3	**PRDX5**	Peroxiredoxin 5	**TPO**	Thyroid peroxidase
**DUSP1**	Dual specificity phosphatase 1	**MPO**	Myeloperoxidase	**PRDX6**	Peroxiredoxin 6	**TTN**	Titin
**EPHX2**	Epoxide hydrolase 2, cytoplasmic	**MPV17**	MpV17 mitochondrial inner membrane protein	**PREX1**	Phosphatidylinositol-3,4,5-trisphosphate-dependent Rac exchange factor 1	**TXNDC2**	Thioredoxin domain containing 2 (spermatozoa)
**EPX**	Eosinophil peroxidase	**MSRA**	Methionine sulfoxide reductase A	**PRG3**	Proteoglycan 3	**TXNRD1**	Thioredoxin reductase 1
**FOXM1**	Forkhead box M1	**MT3**	Metallothionein 3	**PRNP**	Prion protein	**TXNRD2**	Thioredoxin reductase 2
