# Supplementary material for: Calcium Dynamics of Ex Vivo Long-Term Cultured CD8+ T Cells Are Regulated by Changes in Redox Metabolism
Source: PLoS One. 2016 Aug 15;11(8):e0159248. doi: 10.1371/journal.pone.0159248 (PMC4985122; doi:10.1371/journal.pone.0159248)
Supplement: S2 Table — A fold change below 1 corresponds to a downregulation (2-ΔCT). (PDF) [file pone.0159248.s012.pdf]

S2 Table. Exhaustive list of fold changes and their corresponding p-values in targets expressed in CD8+ T cells. A fold change below 1 corresponds to a downregulation ($2^{-\Delta CT}$).

**Symbol**	**Protein Name**	**Fold Change Old/Young**	**p-value**
**PDLIM1**	PDZ and LIM domain 1	0.17	0.01
**ANGPTL7**	Angiopoietin-like 7	2.93	0.01
**MTL5**	Metallothionein-like 5, testis-specific (tesmin)	0.45	0.01
**NUDT1**	Nudix (nucleoside diphosphate linked moiety X)-type motif 1	0.99	0.01
**TTN**	Titin	2.14	0.01
**DUOX1**	Dual oxidase 1	1.68	0.03
**GPX3**	Glutathione peroxidase 3 (plasma)	2.55	0.04
**GLRX2**	Glutaredoxin 2	0.47	0.06
**PRNP**	Prion protein	1.45	0.07
**TXNRD1**	Thioredoxin reductase 1	0.31	0.08
**PRDX1**	Peroxiredoxin 1	0.74	0.13
**CCL5**	Chemokine (C-C motif) ligand 5	3.81	0.15
**TXNRD2**	Thioredoxin reductase 2	1.15	0.18
**GPX2**	Glutathione peroxidase 2 (gastrointestinal)	2.15	0.20
**SEPP1**	Selenoprotein P, plasma, 1	0.34	0.21
**ATOX1**	ATX1 antioxidant protein 1 homolog (yeast)	0.50	0.22
**BNIP3**	BCL2/adenovirus E1B 19kDa interacting protein 3	1.12	0.23
**CCS**	Copper chaperone for superoxide dismutase	0.22	0.23
**PX1**	Glutathione peroxidase 1	0.18	0.23
**PREX1**	Phosphatidylinositol-3,4,5-trisphosphate-dependent Rac exchange factor 1	0.90	0.24
**PRDX5**	Peroxiredoxin 5	0.80	0.29
**CSDE1**	Cold shock domain containing E1, RNA-binding	0.49	0.30
**SIRT2**	Sirtuin 2	1.25	0.32
**FOXM1**	Forkhead box M1	1.78	0.33
**GTF2I**	General transcription factor IIi	0.52	0.34
**GSS**	Glutathione synthetase	0.26	0.37
**NCF1**	Neutrophil cytosolic factor 1	9.32	0.42
**ALOX12**	Arachidonate 12-lipoxygenase	0.60	0.46
**STK25**	Serine/threonine kinase 25	4.65	0.46
**GPX4**	Glutathione peroxidase 4 (phospholipid hydroperoxidase)	0.02	0.47
**SELS**	Selenoprotein S	0.10	0.48
**MPV17**	MpV17 mitochondrial inner membrane protein	0.57	0.49
**MGST3**	Microsomal glutathione S-transferase 3	0.49	0.50
**GSR**	Glutathione reductase	0.73	0.51
**PRDX2**	Peroxiredoxin 2	0.75	0.51
**SOD1**	Superoxide dismutase 1, soluble	6.52	0.51
**SRXN1**	Sulfiredoxin 1	0.12	0.52
**CAT**	Catalase	122.49	0.56
**MSRA**	Methionine sulfoxide reductase A	0.79	0.57
**MT3**	Metallothionein 3	0.28	0.59
**NCF2**	Neutrophil cytosolic factor 2	1.49	0.59

**OXR1**	Oxidation resistance 1	0.02	0.59
**GPX7**	Glutathione peroxidase 7	0.17	0.60
**EPX**	Eosinophil peroxidase	0.83	0.64
**CYBA**	P22-phox	0.41	0.65
**GSTZ1**	Glutathione transferase zeta 1	1.89	0.65
**PRDX6**	Peroxiredoxin 6	3.93	0.65
**IPCEF1**	Interaction protein for cytohesin exchange factors 1	0.65	0.66
**PRDX4**	Peroxiredoxin 4	2.87	0.70
**SOD2**	Superoxide dismutase 2, mitochondrial	0.33	0.70
**DUSP1**	Dual specificity phosphatase 1	4.56	0.71
**RNF7**	Ring finger protein 7	6.55	0.71
**SFTPD**	Surfactant protein D	3.35	0.72
**DHCR24**	24-dehydrocholesterol reductase	0.57	0.82
**PRDX3**	Peroxiredoxin 3	1.60	0.84
**OXSR1**	Oxidation resistance 1	2.20	0.95
**PNKP**	Polynucleotide kinase 3'-phosphatase	1.59	0.98
