# Supplementary material for: Calcium Dynamics of Ex Vivo Long-Term Cultured CD8+ T Cells Are Regulated by Changes in Redox Metabolism
Source: PLoS One. 2016 Aug 15;11(8):e0159248. doi: 10.1371/journal.pone.0159248 (PMC4985122; doi:10.1371/journal.pone.0159248)
Supplement: S3 Table — (PDF) [file pone.0159248.s013.pdf]

S3 Table S3. Normalized mRNA levels of individual genes expressed in young CD8+ T cells, ranked in descending order of expression (n=6).

Symbol	Protein Name	Mean ΔCt ± SEM	% CV
**CYBA**	P22-phox	3.77 ± 0.4	10.64
**CCL5**	Chemokine (C-C motif) ligand 5	3.58 ± 0.68	19.06
**SOD1**	Superoxide dismutase 1, soluble	4.19 ± 0.23	5.56
**GPX4**	Glutathione peroxidase 4 (phospholipid hydroperoxidase)	4.38 ± 0.40	9.24
**PRDX6**	Peroxiredoxin 6	4.60 ± 0.30	6.48
**GTF2I**	General transcription factor IIi	4.79 ± 0.28	5.93
**PRDX3**	Peroxiredoxin 3	4.61 ± 0.46	9.98
**PRDX2**	Peroxiredoxin 2	4.78 ± 0.35	7.24
**GPX1**	Glutathione peroxidase 1	5.17 ± 0.23	4.48
**CSDE1**	Cold shock domain containing E1, RNA-binding	5.19 ± 0.23	4.48
**FOXM1**	Forkhead box M1	5.39 ± 0.18	3.44
**PRDX5**	Peroxiredoxin 5	5.38 ± 0.28	5.25
**ATOX1**	ATX1 antioxidant protein 1 homolog (yeast)	5.60 ± 0.21	3.70
**TXNRD1**	Thioredoxin reductase 1	6.17 ± 0.08	1.33
**DHCR24**	24-dehydrocholesterol reductase	6.38 ± 0.18	2.83
**RNF7**	Ring finger protein 7	6.58 ± 0.21	3.13
**SOD2**	Superoxide dismutase 2, mitochondrial	6.59 ± 0.21	3.12
**OXSR1**	Oxidative-stress responsive 1	6.98 ± 0.10	1.47
**CAT**	Catalase	6.78 ± 0.4	5.92
**PRDX4**	Peroxiredoxin 4	6.99 ± 0.36	5.18
**MGST3**	Microsomal glutathione S-transferase 3	7.18 ± 0.23	3.22
**MPV17**	MpV17 mitochondrial inner membrane protein	7.18 ± 0.23	3.21
**GLRX2**	Glutaredoxin 2	7.39 ± 0.18	2.49
**GSS**	Glutathione synthetase	7.37 ± 0.28	3.81
**BNIP3**	BCL2/adenovirus E1B 19kDa interacting protein 3	7.38 ± 0.34	4.62
**SELS**	Selenoprotein S	7.78 ± 0.28	3.62
**STK25**	Serine/threonine kinase 25	7.98 ± 0.10	1.24
**TXNRD2**	Thioredoxin reductase 2	7.79 ± 0.34	4.43
**NUDT1**	Nudix (nucleoside diphosphate linked moiety X)-type motif 1	8.18 ± 0.09	1.04
**PNKP**	Polynucleotide kinase 3'-phosphatase	7.99 ± 0.31	3.89
**GPX7**	Glutathione peroxidase 7	8.00 ± 0.31	3.89
**OXR1**	Oxidation resistance 1	8.19 ± 0.23	2.85
**PRDX1**	Peroxiredoxin 1	8.19 ± 0.30	3.70
**CCS**	Copper chaperone for superoxide dismutase	8.80 ± 0.19	2.13
**IPCEF1**	Interaction protein for cytohesin exchange factors 1	8.57 ± 0.58	6.78
**MTL5**	Metallothionein-like 5, testis-specific (tesmin)	8.97 ± 0.31	3.46
**SIRT2**	Sirtuin 2	9.01 ± 0.38	4.20
**PREX1**	Phosphatidylinositol-3,4,5-trisphosphate-dependent Rac exchange factor 1	9.18 ± 0.23	2.49
**GSTZ1**	Glutathione transferase zeta 1	9.19 ± 0.37	4.07
**NCF2**	Neutrophil cytosolic factor 2	9.38 ± 0.39	4.20
**NCF1**	Neutrophil cytosolic factor 1	9.00 ± 0.89	9.91

**SRXN1**	Sulfiredoxin 1	10.18 ± 0.23	2.26
**MSRA**	Methionine sulfoxide reductase A	10.18 ± 0.3	2.93
**DUSP1**	Dual specificity phosphatase 1	10.19 ± 0.43	4.19
**GPX3**	Glutathione peroxidase 3 (plasma)	10.37 ± 0.49	4.76
**SEPP1**	Selenoprotein P, plasma, 1	10.57 ± 0.29	2.80
**PRNP**	Prion protein	11.01 ± 0.10	0.90
**GSR**	Glutathione reductase	10.78 ± 0.34	3.17
**PDLIM1**	PDZ and LIM domain 1	10.98 ± 0.3	2.79
**TTN**	Titin	11.19 ± 0.42	3.79
**MT3**	Metallothionein 3	11.4 ± 0.73	6.40
**ANGPTL7**	Angiopoietin-like 7	12.17 ± 0.23	1.87
**ALOX12**	Arachidonate 12-lipoxygenase	12.17 ± 0.42	3.48
**EPX**	Eosinophil peroxidase	12.58 ± 0.41	3.29
**DUOX1**	Dual oxidase 1	12.78 ± 0.34	2.68
**SFTPD**	Surfactant protein D	12.57 ± 0.76	6.07
**PXDN**	Peroxidasin homolog (Drosophila)	13.21 ± 0.62	4.70
**GPX2**	Glutathione peroxidase 2 (gastrointestinal)	13.99 ± 0.47	3.36
