# Supplementary material for: Calcium Dynamics of Ex Vivo Long-Term Cultured CD8+ T Cells Are Regulated by Changes in Redox Metabolism
Source: PLoS One. 2016 Aug 15;11(8):e0159248. doi: 10.1371/journal.pone.0159248 (PMC4985122; doi:10.1371/journal.pone.0159248)
Supplement: S4 Table — (PDF) [file pone.0159248.s014.pdf]

S4 Table. Optimized parameter set obtained from the Jurkat T Cell Model fitting used for the seeding the initial population of parameter values for the genetic algorithm optimization of the Young CD8$^+$ T Cell Model to experimental data.

Parameter	Jurkat T cells
$\boldsymbol{\beta_i}$*	0.056
$\boldsymbol{\beta_{er}}$*	0.049
$\boldsymbol{\beta_{mit}}$	0.033
$\boldsymbol{\rho_{er}}$	0.015
$\boldsymbol{\rho_{mit}}$	0.08
$\boldsymbol{k_{PLCact}}$	0.0033 s^{-1}
$\boldsymbol{k_{PLCdeact}}$	0.042 s^{-1}
$\boldsymbol{k_{IP3prod}}$	0.48 $\mu M^{-1}\ s^{-1}$
$\boldsymbol{k_{IP3deg}}$*	0.010 s^{-1}
$\boldsymbol{V_{IP3}}$	4.0 s^{-1}
$\boldsymbol{K_{IP3}}$	0.57 μM
$\boldsymbol{K_{act}}$	0.13 μM
$\boldsymbol{A}$	0.079
$\boldsymbol{K_{inh}}$	1 μM
$\boldsymbol{K_{IP3inh}}$*	0.82 μM
$\boldsymbol{K_{ERleak}}$*	0.0043 s^{-1}
$\boldsymbol{V_{serca}}$*	112.75 $\mu M\ s^{-1}$
$\boldsymbol{K_{serca}}$	0.43 μM
$\boldsymbol{V_{mitin}}$	388.6 $\mu M\ s^{-1}$
$\boldsymbol{K_{mitin}}$	0.81 μM
$\boldsymbol{V_{mitout}}$*	188.9 $\mu M\ s^{-1}$
$\boldsymbol{K_{mitout}}$*	4.03 μM
$\boldsymbol{V_{crac}}$	2.4 $\mu M\ s^{-1}$
$\boldsymbol{K_{soc}}$*	363.5 μM
$\boldsymbol{K_{stim}}$	178.1 μM

$\boldsymbol{K_{PMleak}}$	1.1e-6 s^{-1}
$\boldsymbol{V_{pmca}}$*	2.14 μM s^{-1}
$\boldsymbol{K_{pmca}}$	0.11 μM
